# Combining datasets for maize root seedling traits increases the power of GWAS and genomic prediction accuracies

**DOI:** 10.1093/jxb/erac236

**Published:** 2022-05-24

**Authors:** Leandro Tonello Zuffo, Rodrigo Oliveira DeLima, Thomas Lübberstedt

**Affiliations:** Corteva Agriscience, Rio Verde, GO, Brazil; Department of Agronomy, Universidade Federal de Viçosa, Viçosa, MG, Brazil; Department of Agronomy, Iowa State University, Ames, IA, USA; Department of Agronomy, Universidade Federal de Viçosa, Viçosa, MG, Brazil; Department of Agronomy, Iowa State University, Ames, IA, USA

**Keywords:** Association mapping, candidate genes, genomic selection, inbred line panel, linkage disequilibrium, population structure, *Zea mays* L

## Abstract

The identification of genomic regions associated with root traits and the genomic prediction of untested genotypes can increase the rate of genetic gain in maize breeding programs targeting roots traits. Here, we combined two maize association panels with different genetic backgrounds to identify single nucleotide polymorphisms (SNPs) associated with root traits, and used a genome-wide association study (GWAS) and to assess the potential of genomic prediction for these traits in maize. For this, we evaluated 377 lines from the Ames panel and 302 from the Backcrossed Germplasm Enhancement of Maize (BGEM) panel in a combined panel of 679 lines. The lines were genotyped with 232 460 SNPs, and four root traits were collected from 14-day-old seedlings. We identified 30 SNPs significantly associated with root traits in the combined panel, whereas only two and six SNPs were detected in the Ames and BGEM panels, respectively. Those 38 SNPs were in linkage disequilibrium with 35 candidate genes. In addition, we found higher prediction accuracy in the combined panel than in the Ames or BGEM panel. We conclude that combining association panels appears to be a useful strategy to identify candidate genes associated with root traits in maize and improve the efficiency of genomic prediction.

## Teaser

Combining inbred line panels is a useful strategy to identify candidate genes associated with root traits in maize and improve the efficiency of the genomic prediction in maize breeding programs.

## Introduction

Maize is one the most important cereal crops, with a wide geographic distribution (Hake and Ross-Ibarra, 2015; Andorf *et al.*, 2019). It is also the cereal with the highest global production, reaching 1187 million tons during the 2020–2021 crop season (USDA, 2021). It has a sophisticated and complex root architecture that is important for plant anchorage and uptake of nutrients and water (Lynch, 2013). Root growth and demand for nutrients are closely associated with grain yield (Cai *et al.*, 2012; Abdel-Ghani *et al.*, 2013; Mu *et al.*, 2015), nitrogen use efficiency (York and Lynch, 2015; Yu *et al.*, 2015; Torres *et al.*, 2019), and phosphorus acquisition (Maharajan *et al.*, 2018; Sun *et al.*, 2018). This unique root architecture allows an efficient uptake of nutrients and water from soils as a prerequisite for high yields and yield stability (Rogers and Benfey 2015; Hochholdinger *et al.*, 2018).

Root-related traits are difficult and laborious to measure, and thus the identification of genes underlying root traits may help maize breeders to use marker-assisted selection for highly efficient root systems and, consequently, for developing maize varieties that are more efficient in nutrient use and tolerant to drought. Genome-wide association study (GWAS) is a powerful tool for gene mapping in plants and animals, and has been widely used for genetic dissection of complex quantitative traits in many major crop species (Xiao *et al.*, 2017; Liu and Yan, 2019; Cortes *et al.*, 2021). In maize, GWAS has been successfully employed to understand the genetic basis of, and to identify candidate genes related to, flowering time and plant height (Vanous *et al.*, 2018; Li *et al.*, 2020; Zheng *et al.*, 2021a), stalk traits (Zhang *et al.*, 2018; Mazaheri *et al.*, 2019), tassel architecture (Wu *et al.*, 2016; Y. Wang *et al.*, 2019), nitrogen use efficiency (Morosini *et al.*, 2017; Ertiro *et al.*, 2020), low-phosphorus tolerance (Xu *et al.*, 2018; Q. J. Wang *et al.*, 2019), cold tolerance (Yi *et al.*, 2021), drought tolerance (Zaidi *et al.*, 2016; Yuan *et al.*, 2019; Liu and Qin, 2021), grain yield and yield-related traits (Zhang *et al.*, 2020a; Yang *et al.*, 2020; Ma and Cao, 2021), and grain quality (Zhang *et al.*, 2020b; Zheng *et al.*, 2021b; Zhou *et al.*, 2021). Moreover, several candidate genes involved in genetic control of root-related traits have been identified in maize by a GWAS approach. Ma *et al.* 2020) carried out a GWAS across 226 maize doubled haploid introgression lines under contrasting nitrogen lwevels and found 68 and 48 candidate genes associated with root architecture traits under low and high nitrogen, respectively. Using 80 elite inbred lines in a GWAS, Moussa *et al.* (2021) identified 46 candidate genes associated with maize root morphological traits, and five promising genes that regulate root branching were verified for expression level. In another recent study, Sun *et al.* (2021) evaluated an association panel of 461 maize inbred lines across two nitrogen levels and detected respectively 33 and 22 candidate genes associated with root traits. Thus, GWAS has great potential to identified molecular markers associated with candidate genes underlying variation in maize root architecture traits.

In the past, breeders selected individuals based on phenotypic selection and pedigree information. With advances in molecular genetics, genomic prediction has become a promising method to shorten the breeding cycle, reduce the costs per cycle, and save labor costs (Wang *et al.*, 2018). Pace *et al.* (2015b) used genomic prediction on a subset of 384 inbred lines from the Ames panel and found average prediction accuracies between 0.38 and 0.55 for root seedling traits. Cantelmo *et al.* (2017) evaluated 838 and 797 single-cross hybrids for grain yield in winter and summer seasons, respectively, in different locations to estimate the prediction accuracy using different training and validation populations. They found ranges of correlations of 0.82–0.89 in the winter season, 0.56–0.76 in the summer season, and 0.53 between different seasons and locations.

Advances in genotyping technology, in particular genotyping-by-sequencing (GBS) methods, have led to a considerable reduction in costs for genotyping a large number of individuals with high density single nucleotide polymorphism (SNP) markers (Poland and Rife, 2012). Hence, many association mapping panels in maize with different genetic background have been genotyped and phenotyped for several traits around the world, and most of them are public panels that are available for use by the scientific community. The maize nested association mapping (NAM) population is a collection of 5000 recombinant inbred lines from 26 diverse founders (Yu *et al.*, 2008), and the Ames panel is a collection of 2815 inbred lines from breeding programs around the world (Romay *et al.*, 2013). Thus, information across panels and traits can be combined using appropriate statistical models to exploit the genetic architecture of complex traits in maize (Yang *et al.*, 2011). In soybean, Torkamaneh and Belzile (2015) combined two SNP datasets derived from GBS and a SNP array (SoySNP50K) from 139 soybean accessions previously phenotyped for seed oil content to perform a GWAS analysis. They found that the number of significant marker–trait associations and significance levels increased considerably when using the combined dataset.

In our study, we combined two datasets from previous GWAS studies with a different genetic background to perform GWAS and genomic selection using several scenarios. Our hypothesis was that combining the genomic data from different backgrounds increases the power of detecting polymorphisms associated with maize seedling root traits, and improving the prediction accuracy across different datasets is possible. Thus, our objectives were to: (i) identify the genomic regions associated with root traits in maize in a combined panel including two association panels and assess the effect of combining datasets in GWAS, and (ii) assess the potential of genomic prediction in two maize inbred line panels *per se* and in the combined panel using different sets of training and validation populations and several genetic models for root traits in maize.

## Material and methods

### Plant materials

We evaluated 679 maize inbred lines from two association panels: 377 inbred lines from the Ames panel (Romay *et al.*, 2013) and 302 doubled haploid (DH) lines from the Backcrossed Germplasm Enhancement of Maize (BGEM) panel (Supplementary Table S1; Sanchez *et al.*, 2018). The Ames panel consists of a collection of 2815 maize inbred lines conserved at USDA-ARS North Central Regional Plant Introduction Station in Ames, IA, USA (Romay *et al.*, 2013). The BGEM panel was constructed as follows: a backcross (BC_1_F_1_) population was developed using exotic maize landraces as donors and two expired Plant Variety Protection lines, PHZ51 and PHB47, as recurrent parents, within the Germplasm Enhancement of Maize (GEM) project. BC_1_F_1_ plants were crossed with the inducer hybrids RWS9×RWK-76 to produce haploid plants (Röber *et al.*, 2005; Liu *et al.*, 2016). Haploid seeds were planted in greenhouse, and seedlings were treated with colchicine to promote genome doubling. Then, they were transplanted to the field to produce DH lines (Sanchez *et al.*, 2018).

### Phenotypic data

The 377 inbred lines from the Ames panel and 302 DH lines from the BGEM panel were previously characterized for root morphological traits by Pace *et al.* (2015a) and Sanchez *et al.* (2018), respectively, using the protocol for root phenotyping and image analysis described by Pace *et al.* (2014). Briefly, four seeds of each inbred line and DH inbred line were grown in rolled germination papers, and each paper roll with four seedlings was considered an experimental unit. The experiments were carried out in growth chambers under controlled conditions, and both the Ames and BGEM panels were grown in a randomized block design with three independent replicates per line. After 14 d, seedlings were removed from the growth chamber and all root traits were measured. Then, the seedling phenotypes were obtained by image analysis using the software ARIA (Automatic Root Image Analyzer; Pace *et al.*, 2014). In our study, we used four root morphological traits: primary root length (PRL, cm), lateral root length (LRL, cm), total root length (TRL, cm), and total number of roots (TNR).

### Phenotypic data analysis

A mixed model implemented in the R package ‘lme4’ (Bates *et al.*, 2015) was used to estimate the variance components and to predict the genotypic values of each line in the Ames and BGEM panels. Line was considered as a random effect and replication was included in the model as a fixed effect. Variance components were estimated by using a restricted estimation of maximum likelihood, and genotypic values of inbred and DH lines were predicted using the best linear unbiased predictors (BLUPs; Piepho *et al.*, 2008). A likelihood ratio test deviance analysis was used to test random effects via the chi-square statistic (Resende *et al.*, 2007). Ranges and mean values were based on BLUPs. Broad-sense heritability (${\hat{h}}_{\bar{X}}^{2}$ on a line-mean basis was estimated for each trait in both panels as follows (Hallauer *et al.*, 2010):

${\hat{h}}_{\bar{X}}^{2}=\frac{{\hat{\sigma }}_{G}^{2}}{{\hat{\sigma }}_{G}^{2}+\frac{{\hat{\sigma }}^{2}}{r}}$ ,

where ${\hat{\sigma }}_{G}^{2}$ and ${\hat{\sigma }}^{2}$ are genotypic variance estimates due to lines and error variance estimates, respectively, and *r* is the number of replications.

### Genotypic data

The Ames panel was originally genotyped on build version ZeaGBSv1.0, but to compare both panels we used the latest build version, ZeaGBSv2.7 (Elshire *et al.*, 2011), with 955 690 GBS markers generated at the Cornell Institute for Genomic Diversity laboratory where the BGEM panel was genotyped and aligned with B73 AGPv2 coordinates as reference genome.

The genotypic data from the Ames and BGEM panels were merged into one dataset, called the Combined panel. Markers with more than 20% of missing data, markers with minor allele frequency lower than 5%, and monomorphic markers were removed. The imputation method used was linkage disequilibrium *K*-number neighbor imputation (Money *et al.*, 2015). All before-mentioned steps were conducted in Tassel 5.0 (Bradbury *et al.*, 2007). The final number of markers used for further analysis was 232 460 SNPs, distributed across all chromosomes with 0.01% of missing data and an average heterozygosity of 0.02%.

### Genotypic data analysis

#### Population structure and linkage disequilibrium analysis

The model-based program fastStructure (Raj *et al.*, 2014), a fast algorithm based on a Variational Bayesian framework implemented in the program Structure, was used to infer population structure using 232 460 selected SNP markers. We performed population structure analysis in the Ames, BGEM, and Combined panels with allowed model complexity *K*∈{1, …, 9}, where *K* denotes the number of populations. We set a convergence criterion of 10 × 10^−6^ (when the increase in log likelihood is <10 × 10^−6^), logistic prior, and five cross-validations. To identify the *K* that explained the population structure most, we followed the recommendation of Raj *et al.* (2014) with the value of *K* that maximizes the log-marginal likelihood of the entire dataset.

In the Combined panel, linkage disequilibrium (LD) between pairs of SNPs for each chromosome was determined using squared Pearson correlation coefficients (*r*^2^) between vectors of SNP alleles following Hill and Weir (1988). The LD decay cutoff *r*^2^ value was set to be 0.2. The TASSEL 5.0 package was run with a 50 kb sliding window, which determines the width of the window on one side of the start, and LD was calculated for sites within the window for sites surrounding the current site using 232 460 SNP markers evenly distributed across all chromosomes (Bradbury *et al.*, 2007). Thus, the spacing between two loci on the same chromosome was segmented in 50 kb and the average LD was assessed for each window.

#### Genome-wide association analysis

Best unbiased prediction (BLUP) estimates of lateral root length, primary root length, total number of roots, and total root length were used for GWAS in each panel and in the Combined panel. Although GWAS has been previously conducted in the Ames (Pace *et al.*, 2015a) and BGEM (Sanchez *et al.*, 2018) panels, we carried out GWAS in the both panels again because their genotyped data were based on a different build version of ZeaGBS. For two individual panels and the Combined panel, GWAS was performed using the FarmCPU (Fixed and random model Circulating Probability Unification) model implemented in the R package GAPIT (Lipka *et al.*, 2012). The FarmCPU model controls false positives and prevents over-fitting by applying algorithms that resolve confounding problems among testing markers and covariates (Liu *et al.*, 2016). We used the Q matrix from fastStructure in the model and calculated the kinship (K) matrix using VanRaden’s method (VanRaden, 2008; Raj *et al.*, 2014). Employing the Q+K matrix improves statistical power and reduces spurious associations (Yu *et al.*, 2006). To determine the significance threshold for multiple testing and to reduce type I error, the statistical program Simple*M* was applied (Gao *et al.*, 2008). Simple*M* calculates a composite LD (CLD) correlation matrix from SNP genotypes, and a correlation matrix for all markers with the corresponding eigenvalues for each SNP locus (*Meff*_G). The effective number of independent tests is calculated, and applied similarly to the Bonferroni correction method (α/*Meff*_G). We set the α-threshold at 0.05. The significant SNPs were obtained by comparing the *P*-value from FarmCPU analysis and the Simple*M* threshold. The LD decay distance (*r*^2^=0.2) for each chromosome was used to determine the size of the chromosome segment containing a significant SNP, to search for candidate genes. The MaizeGDB database was used to find linked candidate genes for each SNP based on the maize B73 RefGen_V2 genome, and to find matches with B73 RefGen_V4. With the gene ID, Ensembl Biomart (Kinsella *et al.*, 2011) was used to obtain information of the candidate genes.

#### Genomic-wide prediction

Genomic estimated breeding values (GEBVs) were calculated for 11 scenarios (Table 1). Accuracy was defined as the correlation between predicted and observed phenotypic values. Our strategy was based on fastStructure subpopulation results. First, we estimated GEBVs within each panel (Ames, BGEM, and Combined) using cross-validation. We randomly attributed 60% of lines as the training set and 40% as the validation set and repeated this procedure five times. Then, we estimated GEBVs across the Ames and BGEM panels, using one as training and the other as validation set, and vice versa. After that, we made predictions across the different subpopulations of the Ames and BGEM panels. Finally, we estimated GEBVs using each subpopulation of the Combined panel using cross-validation, and we randomly assigned 60% of lines as the training set and 40% as the validation set, repeated five times.

**Table 1.** Summary of the 11 scenarios used for training and validation dataset for genome wide selection analysis

Scenario^a^	SS BGEM^b^	NSS BGEM	SS Ames	NSS Ames	Training^c^	Validation^d^
Ames	—	—	39	188	227	150
BGEM	101	79	—	—	180	122
Ames→BGEM	—	—	65	314	379	300
BGEM→Ames	169	131	—	—	300	379
NSS Ames→NSS BGEM	—	—	—	314	314	131
NSS BGEM→NSS Ames	—	131	—	—	131	314
SS Ames→SS BGEM	—	—	65	—	65	169
SS BGEM→SS Ames	169	—	—	—	169	65
NSS Combined	—	79	—	188	267	178
SS Combined	101	—	39	—	140	94
Combined	101	79	39	188	407	272

Training set before the arrow, and validation set after the arrow.

Number of lines used on the training set in each subpopulation from fastStructure in each scenario. SS and NS refer to Stiff Stalk and non-Stiff Stalk subpopulation, respectively.

Total number of lines used as training set in each scenario.

Total number of lines used as validation set in each scenario.

In addition to strategies involving the 11 scenarios to estimate the GEBVs, we considered four genetic models for genomic prediction that differed mainly in their priors at α, i.e. how markers were incorporated in the models. We used Ridge regression-best linear unbiased prediction (RR-BLUP) in the R package rrBLUP (Endelman, 2011), and BayesA, BayesB, and BayesC in the R package BGLR (Pérez and de los Campos 2014) using 30 000 iterations, including a burn-in period of 5000. All models followed the basic model: *y*=*X*β+*Z*α+ε, where *y* is a vector of phenotypes; β is a vector of non-genetic fixed effects; *X* is an incidence matrix for the fixed effects; α is a vector of random regression coefficients of all marker effects; *Z* is an *n*×*k* genotypic matrix of markers; and ε is a vector of residuals. The four models are explained in detail elsewhere (Wang *et al*., 2015, 2018; Crossa *et al.*, 2017). Briefly, the model RR-BLUP has a marginal multivariate *t* distribution with mean zero, and all markers with the same variance. BayesA has higher mass at zero that induces shrinkage of estimates that is size-of-effect dependent, and the prior used is a scaled-*t* distribution. BayesB and BayesC are a mixture of priors that are similar in point of mass at zero and differ in a scaled-*t* and Gaussian distribution, respectively (Gianola, 2013).

## Results

### Phenotypic variation and estimates of broad-sense heritability

The traits followed a normal distribution with a slight skew to the left for LRL and to the right for PRL. Considerable variation was observed among lines for all five root morphological traits in both the Ames and the BGEM panel (Table 2). However, the DH lines from the BGEM panel showed larger ranges and means than the inbred lines from the Ames panel for root traits. For example, TRL ranged from 128.66 cm to 475.35 cm (mean=263.42 cm) and from 69.26 cm to 323.24 cm (mean=174.26 cm) in the BGEM and Ames panel, respectively; and TRN ranged from 6.98 to 23.87 roots (mean=13.74) and from 3.84 to 17.56 roots (mean=10.50) in the BGEM and Ames panel, respectively. The largest standard deviations were found for TRL: 53.1 and 49.7 cm for the BGEM and Ames panel, respectively. Estimates of broad-sense heritability $\left({\hat{h}}_{\bar{X}}^{2}\right)$ were slightly higher in the Ames than in the BGEM panel for all traits. The $\left({\hat{h}}_{\bar{X}}^{2}\right)$ values were low-to-intermediate and they ranged from 0.28 (PRL) to 0.49 (TNR), and from 0.24 (PRL) to 0.45 (TRN) in the Ames and BGEM panels, respectively.

**Table 2.** Best linear unbiased prediction estimates of means, standard deviation (SD), minimum and maximum, and estimates of broad-sense heritability $\left({\hat{h}}_{\bar{X}}^{2}\right)$ for primary (PRL, cm), lateral (LRL, cm) and total (TRL, cm) root length, and total number of roots (TRN) measured in 302 and 377 maize inbred lines from BGEM and Ames panels, respectively

Trait	BGEM panel					Ames panel				
	Mean	SD	Min	Max	${\hat{h}}_{\bar{X}}^{2}$	Mean	SD	Min	Max	${\hat{h}}_{\bar{X}}^{2}$
PRL	32.16	2.40	22.67	38.59	0.24	27.42	3.27	17.07	38.26	0.28
LRL	231.25	51.69	107.63	438.52	0.38	134.32	47.56	36.23	283.64	0.42
TRL	263.42	52.95	128.66	475.35	0.37	174.26	49.70	69.26	323.24	0.42
TNR	13.74	2.97	6.98	23.87	0.45	10.50	2.86	3.84	17.56	0.49

### Linkage disequilibrium and population structure

**Fig. 1.**
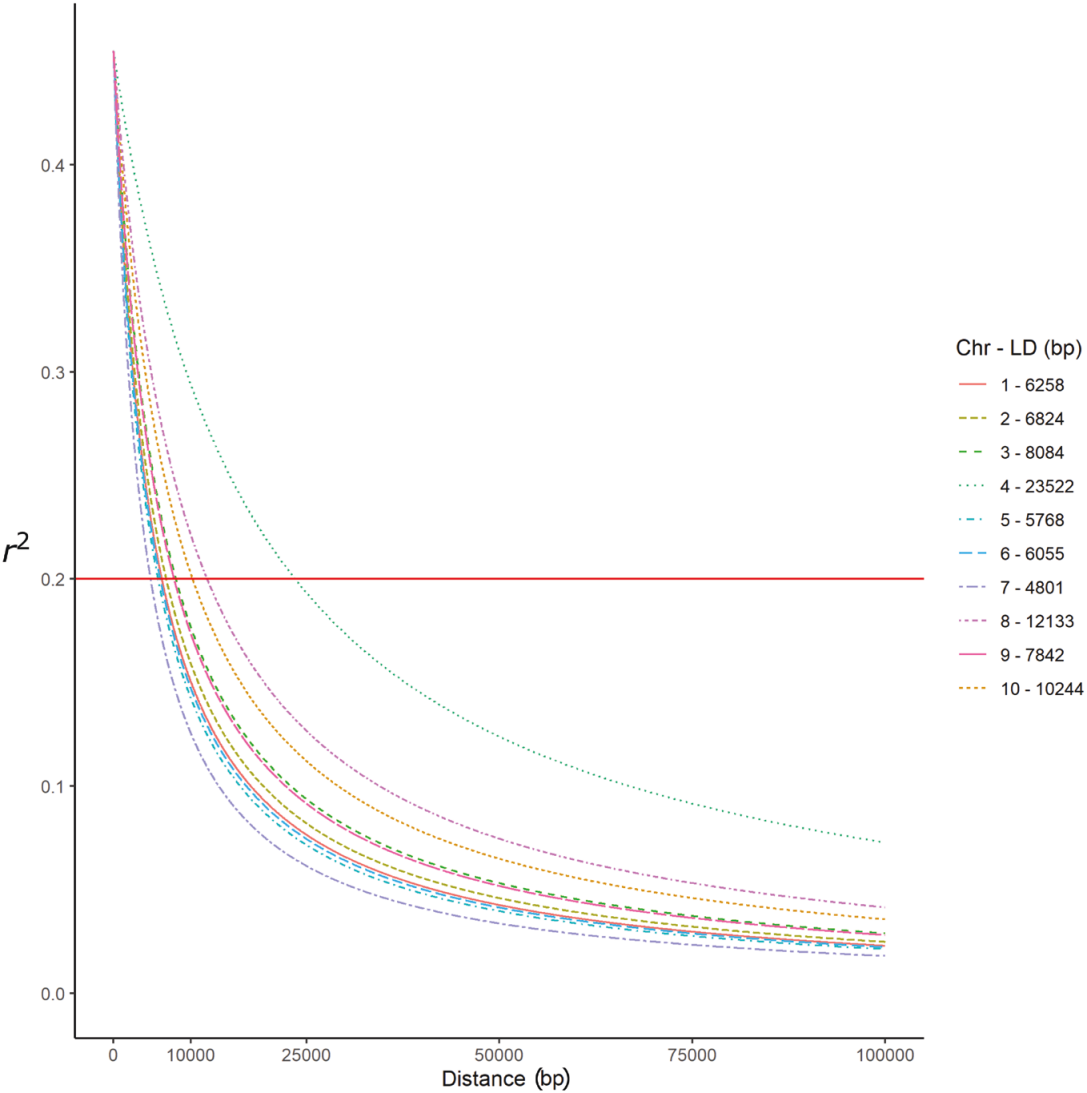
Whole-genome linkage disequilibrium decay in the entire 679 maize lines from the Combined panel. Linkage disequilibrium within and over chromosomes is given in physical distances of 50 kb.

Linkage disequilibrium among the 679 maize lines in the Combined panel was measured for all 232 460 markers across the 10 chromosomes. The average LD decay was 9 kb, and was similar across the 10 chromosomes, except for chromosome 4, which showed the slowest decay of approximately 23 kb (Fig. 1). The range of LD decay was 4801 bp (chromosome 7) to 23 522 bp (chromosome 4).

**Fig. 2.**
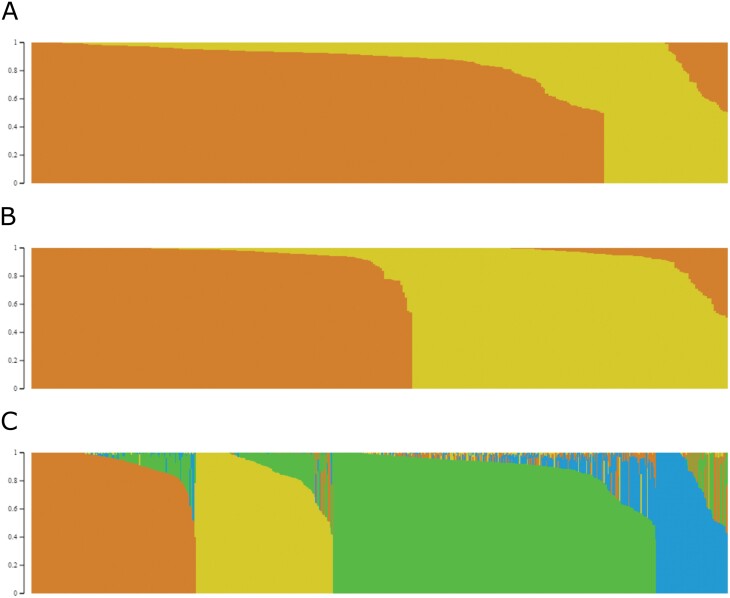
Analysis of the population structure of 377 inbred lines from Ames panel (A), 302 DH lines from BGEM panel (B), and 679 maize lines from the Combined panel (C) using SNP markers. fastStructure clustering results obtained at *K*=4. Each inbred line is represented by a thin bar corresponding to the sum of assignment probabilities to the *K* cluster. For Ames and BGEM panels, orange and yellow refer to NSS and SS subpopulations, respectively; for the Combined panel the association between colors and subpopulations are as follows: orange, SS from BGEM; yellow, NSS from BGEM; blue, SS from Ames; green, NSS from Ames. Panel A (population structure results from Ames panel) was adapted from Pace *et al.* (2015b).

In a population structure analysis using the model-based program fastStructure, the most significant peak of Δ*K* was observed when *K*=2 for both panels, and the 377 inbred lines from Ames and 302 DH lines from BGEM were clustered into two subpopulations each (Fig. 2). According to information of pedigree and heterotic groups, the first subpopulation of Ames and BGEM comprised 310 and 137 non-Stiff Stalk (NSS) lines, and 67 and 165 Stiff Stalk (SS) lines, respectively. In the Combined panel, the 679 maize lines clustered into four subpopulations (*K*=4), and the population stratification was consistent with their association panel (BGEM and/or Ames) and heterotic groups in maize. Thus, the first subpopulation comprised 314 inbred lines, mainly NSS from the Ames panel, and some tropical, popcorn, unclassified lines, and Mo17; the second subpopulation comprised 65 mostly SS lines from the Ames panel including B73; and the third and fourth subpopulations comprised 169 PHB47-derived lines and 131 PHZ51-derived lines, respectively, all of them from the BGEM panel. The maize inbred lines PHB47 and PHZ51 are known lines that belong to SS and NSS heterotic groups, respectively. Some BGEM-DH lines were misgrouped into the opposite heterotic group because of high donor (exotic) parent contributions of 60% (Sanchez *et al.*, 2018).

### Genome-wide association studies

**Fig. 3.**
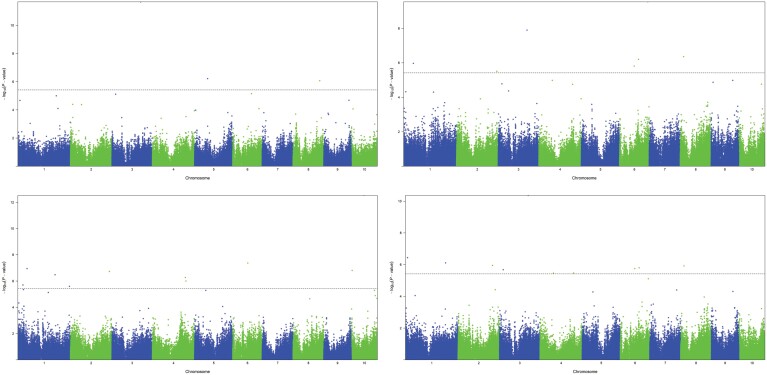
Manhattan plot showing associations between SNP markers and root traits plotted from the association analysis. Lateral root length (A), primary root length (B), total number of roots (C), and total root length (D) in the Combined panel. The dashed horizontal line depicts the simple*M* correction.

The threshold calculated by Simple*M* was different for each panel, and the cutoff was 3.73 × 10^−6^ for the Ames panel and 3.83 × 10^−6^ for the Combined panel. In GWAS for each panel, we found two significant (*P*<3.73 × 10^−6^) SNPs associated with TNR in the Ames panel and six SNPs significantly (*P*<1.76 × 10^−6^) associated with root traits in the BGEM panel: one with TRL, two with LRL, and three with TNR (Table 3). When we combined the panels, we found 30 significantly (*P*<3.73 × 10^−6^) associated SNPs with PRL (10), LRL (7), TNR (3), and TRL (10) (Fig. 3). Those 38 SNPs were associated with 35 candidate genes and 29 unique genes (Table 4). Candidate genes were only considered when trait-associated SNPs were identified within their gene regions.

**Table 3.** Number of SNP markers significantly associated with some root trait in Ames, BGEM, and Combined panels

Trait	Ames	BGEM	Combined
Primary root length (cm)	0	0	10
Lateral root length (cm)	0	2	7
Total root length (cm)	0	1	10
Total number of roots	2	3	3

**Table 4.** Significant SNP markers and candidate genes associated with lateral root length (LRL), primary root length (PRL), total root length (TRL), and total number of roots (TNR) on Ames, BGEM and Combined panel

Panel	Trait	SNP	B73 Gene ID^a^	Zm Gene ID^b^	Function^c^
Ames	TNR	S9_105426105	GRMZM2G331015	Zm00001d046883	Diphthamide biosynthesis protein 3
Ames	TNR	S9_105426105	GRMZM2G031370	Zm00001d046882	Ypt/Rab-GAP domain of gyp1p superfamily protein
BGEM	LRL	S2_229011896	GRMZM5G875516	Zm00001d007603	NB-ARC domain containing protein
BGEM	LRL	S8_3886189	GRMZM2G153434	Zm00001d008285	PQ-loop repeat family protein/ transmembrane family protein
BGEM	TNR	S1_295347415	GRMZM2G127309	Zm00001d034729	Carbohydrate-binding X8 domain superfamily protein
BGEM	TNR	S2_226393146	GRMZM2G106655	Zm00001d007504	Sterol 14-demethylase
BGEM	TNR	S5_212654036	GRMZM5G878379	Zm00001d018326	MAP kinase kinase4
BGEM	TRL	S8_3886189	GRMZM2G153434	Zm00001d008285	PQ-loop repeat family protein/ transmembrane family protein
Combined	LRL	S1_55575549	GRMZM2G072892	Zm00001d029053	Lung seven transmembrane receptor family protein
Combined	LRL	S2_229287344	GRMZM2G333448	Zm00001d007624	Uncharacterized
Combined	LRL	S3_164391691	GRMZM2G357926	Zm00001d042434	Late embryogenesis abundant (LEA) hydroxyproline-rich glycoprotein family
Combined	LRL	S6_107885930	GRMZM2G392700	Zm00001d037109	Smr domain containing protein
Combined	LRL	S6_160702812	GRMZM2G474546	Zm00001d038809	Protein kinase superfamily protein
Combined	LRL	S6_83217874	GRMZM2G132644	Zm00001d036386	Transducin/WD40 repeat-like superfamily protein
Combined	LRL	S8_19122996	GRMZM2G125020	Zm00001d008782	Protein MARD1
Combined	PRL	S1_29197616	GRMZM2G476914	Zm00001d028294	Ubiquitin-protein ligase
Combined	PRL	S1_52765942	GRMZM2G145008	Zm00001d028967	Probable ADP-ribosylation factor GTPase-activating protein AGD14
Combined	PRL	S2_222724929	GRMZM2G094978	Zm00001d007366	Haloacid dehalogenase-like hydrolase domain-containing protein
Combined	PRL	S4_188518876	GRMZM2G011919	Zm00001d052544	Putative CBL-interacting protein kinase family protein
Combined	PRL	S4_191579817	GRMZM2G004699	Zm00001d052651	Probable xyloglucan endotransglucosylase/hydrolase protein 5
Combined	PRL	S6_86338227	GRMZM2G082874	Zm00001d036454	Plant-specific domain TIGR01589 family protein
Combined	PRL	S10_5757204	GRMZM2G113073	Zm00001d023434	Rac GTPase activating protein 1
Combined	PRL	S10_73160806	GRMZM2G343519	Zm00001d024469	Glutaredoxin family protein
Combined	TNR	S3_164391691	GRMZM2G357926	Zm00001d042434	Late embryogenesis abundant (LEA) hydroxyproline-rich glycoprotein family
Combined	TNR	S8_152319365	GRMZM2G487776	Zm00001d011637	Hydroxyproline-rich glycoprotein family protein
Combined	TRL	S1_12165882	GRMZM2G076062	Zm00001d027731	Evolutionarily conserved C-terminal region 2
Combined	TRL	S1_229753355	GRMZM2G413853	Zm00001d032655	Dicer-like 102
Combined	TRL	S3_164391691	GRMZM2G357926	Zm00001d042434	Late embryogenesis abundant (LEA) hydroxyproline-rich glycoprotein family
Combined	TRL	S3_21565733	GRMZM2G309897	Zm00001d039967	Probable leucine-rich repeat receptor-like protein kinase
Combined	TRL	S4_191764922	GRMZM2G142344	Zm00001d052655	SNARE associated Golgi protein family
Combined	TRL	S4_191764922	GRMZM2G142342	Zm00001d052654	Germin-like protein subfamily 3 member 4
Combined	TRL	S4_76384998	GRMZM2G066599	Zm00001d050294	VQ motif-containing protein
Combined	TRL	S6_107885930	GRMZM2G392700	Zm00001d037109	Smr domain containing protein
Combined	TRL	S6_83217874	GRMZM2G132644	Zm00001d036386	Transducin/WD40 repeat-like superfamily protein
Combined	TRL	S8_19122996	GRMZM2G125020	Zm00001d008782	Protein MARD1

Based on B73 RefGen_v2.

Based on B73 RefGen_v4.

Obtained from MaizeGDB and Gramene.

The traits LRL and TRL shared common SNPs. SNP S8_3886189 is linked to gene Zm00001d008285. It codes for a PQ-loop repeat family protein/transmembrane family protein, identified in the BGEM panel. In the Combined panel, we found various candidate genes. SNP S8_19122996 is linked to Zm00001d008782, which encodes protein MARD1 (mediator of ABA-regulated dormancy); S6_83217874 is linked to Zm00001d036386, coding for a Transducin/WD40 repeat-like superfamily protein; and S6_107885930 is linked to gene Zm00001d037109, encoding a Smr (small MutS-related) domain containing protein. SNP S3_164391691 was found in the Combined panel and it was linked to the gene Zm00001d042434, coding for a member of the Late embryogenesis abundant (LEA) hydroxyproline-rich glycoprotein family. This SNP was associated with LRL, TNR, and TRL.

### Genomic-wide prediction

**Fig. 4.**
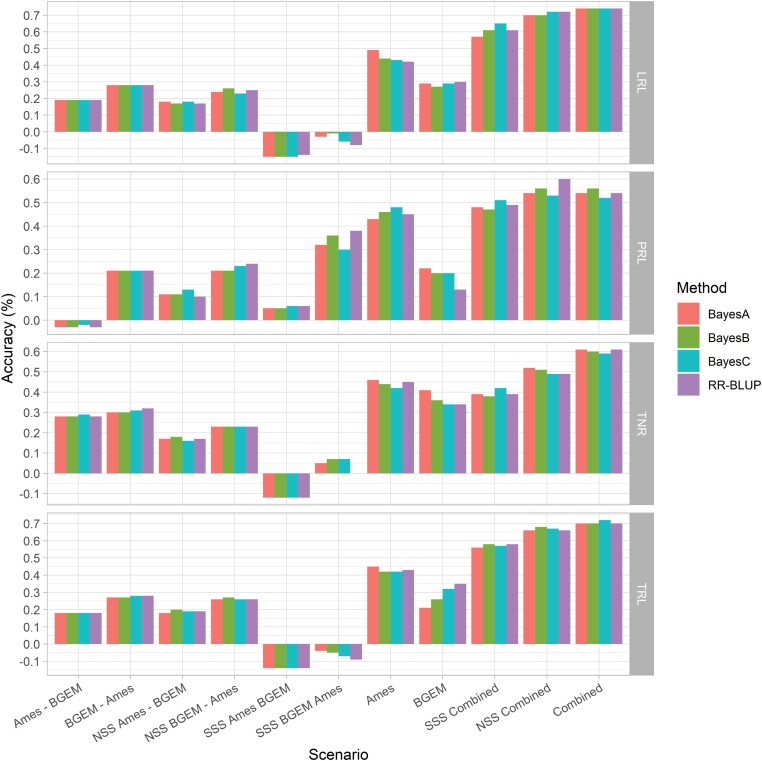
Accuracy assessment of genome wide studies for five root traits, four genetic models and 11 scenarios. LRL: lateral root length; PRL: primary root length; TRL: total root length; TNR: total number of roots.

The GEBVs in the Combined panel showed higher prediction accuracies than when each panel, BGEM or Ames, was analysed separately for all tested root traits and genetic models (Fig. 4). The accuracies for each scenario across traits and genetic models supported that combining datasets improves accuracies. The Combined scenario had the highest accuracy (0.65) across all traits, followed by NSSS Combined (0.61) and BSSS Combined (0.52). The Ames panel showed a higher accuracy than the BGEM panel with 0.44 and 0.28, respectively. When we compared the prediction accuracies of non-combined line sets, we found low prediction accuracies on average (all prediction accuracies were below 0.25), and the scenarios that used only BSSS lines (BGEM or Ames) showed the lowest accuracies. Considering different genetic models, the overall average across traits and scenarios was 0.30.

Comparing the prediction accuracies among traits, generally PRL showed a lower accuracy among all traits whereas LRL showed the highest prediction accuracy. The highest accuracy found was 0.74 for LRL in the Combined panel using BayesC, and the lowest accuracy found was −0.15 for LRL in the BSSS Ames→BGEM scenario using BayesC. For TRL, the highest accuracy was 0.72 in the Combined panel using BayesC. For TNR, the highest accuracy was 0.61 in the Combined panel using RR-BLUP. Concerning PRL, the highest accuracy found was 0.60 in the NSSS Combined scenario using RR-BLUP.

## Discussion

In our study, we combined two maize association panels previously characterized for root architecture traits: the Ames and BGEM panels (Pace *et al.* 2015a; Sanchez *et al.*, 2018). The Ames panel is a subset of 377 temperate maize inbred lines selected from a collection of 2815 inbred lines based on maturity, genetic diversity, and geographic origin, whereas DH lines from BGEM were derived from backcrosses (BC_1_F_1_) among 66 tropical maize landraces and expired-PVP temperate lines, and had about 20% of donor (tropical germplasm) parent contribution (Sanchez *et al.*, 2018). Thus, the larger genotypic variation and means observed in the DH lines from the BGEM panel compared with inbred lines from the Ames panel for all root traits may be due to their genetic background since tropical maize germplasm has greater genetic diversity and tolerance to abiotic stress, which might require more vigorous roots, than temperate germplasm (Lu *et al.*, 2011; Sharma and Carena, 2012; Laude and Carena, 2015).

### Population structure and linkage disequilibrium

The analysis of population structure is important to reduce spurious associations and allocate the individuals into groups for breeding programs and genetic studies, especially if information about pedigree and origins is lacking (Flint-Garcia *et al.*, 2003; Viana *et al.* 2013; Cortes *et al.*, 2021). Torkamaneh *et al.* (2021) used fastStructure to group 1007 soybean accessions into 12 subpopulations that reflect the geographic origin using whole-genome SNP data. Adu *et al.* (2019) studied 94 tropical maize inbred lines using cluster and population structure analysis and concluded that the inbred lines belonged to three heterotic groups, and that results from population structure were more reliable than those obtained by cluster analysis. In another recent study, Faria *et al.* (2022) also used population structure analysis for assigning 182 maize breeding inbred lines with complex and mixed genetic background into three major groups and concluded that they must be explored as three heterotic groups in a maize breeding program. In our study, the population structure results revealed that 679 maize lines from the Combined panel may be placed into four well-defined groups, which was in agreement with the structure of individual association panels and heterotic group affiliations (Fig. 2). Our results agree with those reported by Pace *et al.* (2015a) who used the STRUCTURE model and stratified 384 lines from the Ames panel into two subpopulations: one larger group of 319 lines including Mo17, and another smaller subpopulation with mostly SS lines including B73. In our study, the third group contained BGEM lines with recurrent parent PHB47, a SS inbred line, and the fourth group contained NSS BGEM lines with recurrent parent PHZ51, a known NSS inbred line, as well as PHZ51 from the Ames Panel. Results are consistent with the BGEM subpopulation structure reported by Sanchez *et al.* (2018).

In the present study, the average LD decay across all chromosomes in the Combined panel was close to 9 kb (Fig. 1). This LD decay is consistent with results reported by Dinesh *et al.* (2016), who carried out a genetic diversity and population structure analysis among 64 CIMMYT maize inbred lines using SNP markers from GBS and found the LD decay ranged from 4.31 kb to 15.88 kb across chromosomes at *r*^2^=0.2. In our study, chromosome 4 showed a relatively slow LD decay distance, consistent with Thirunavukkarasu *et al.* (2013), who genotyped 240 subtropical elite maize inbred lines and performed a genetic diversity, population structure, and linkage disequilibrium analysis using SNP markers.

### Genome-wide association studies

The root system is important to extract water and nutrients and to anchor plants in the soil, but it is hard to phenotype root traits. Different methods were developed for a rapid, accurate, and high-throughput screening of root architecture at the seedling stage in the laboratory, such as a paper roll, hydroponic systems, and a sand–vermiculite mixture as growth media (Pace *et al.*, 2014; Torres *et al.*, 2019; Ma *et al.*, 2020; Moussa *et al.*, 2021). To improve breeding success, it is important to integrate genomic tools to identify desirable alleles and use them to access germplasm (Andorf *et al.*, 2019; Cortes *et al.*, 2021; Crossa *et al.*, 2021). In our study, we found 30 SNPs associated with root traits in the Combined panel, and two and six SNPs in the Ames and BGEM panels, respectively (Fig. 3). Interestingly, we observed no overlapping associated SNPs among the individual and combined panels. Since the same biological and marker data, as well as GWAS method (FarmCPU), was used, the ability to detect SNPs must be connected to the altered GWAS population, in terms of increased population size (which increased the power of detecting SNPs associated with some trait), but also altered allele frequencies impacting the power of SNP detection. FarmCPU is a more stringent GWAS method compared with, for example, a general linear model, and resulted in only a few detected SNPs in the smaller populations. As those seemed to not be major QTLs with strong effects, the chance of redetection in the larger panel is limited. If in a larger GWAS population stronger SNP effects are observed elsewhere in the genome, they may mask other SNPs, which may remain below the significance threshold. In older QTL studies, similar observations were made, for example with regard to limited overlap of QTL between different samples of the same populations and explained by limited power of detecting minor QTLs, in particular repeatedly (Lübberstedt *et al.* 1998). As GWAS studies deal with multi-parental populations and lower LD compared with QTL mapping populations, this issue should be increased in GWAS compared with QTL mapping studies.

The identification of molecular markers associated with root traits enables marker-assisted introgression into elite germplasm and increased genetic gain in the breeding programs. Most candidate genes identified in our study were associated with root growth and development in maize. The gene Zm00001d028967 found for primary root length belongs to the ADP-ribosylation factor (ARF) family, which regulates different cellular processes such as cell growth and cell proliferation (Hall, 1990). Jincheng-Yuan *et al.* (2015) isolated an ADP-ribosylation factor in maize, and analysis by real-time quantitative PCR showed higher expression levels in root and embryo than in mature leaves, silks, and seeds. The candidate gene Zm00001d042434 was found for LRL, TNR, and TRL in the Combined panel. It is linked to the LEA gene family in maize and associated with tolerance of abiotic stress. Li and Cao (2016) identified LEA genes in maize and found expression across different tissues examined; the LEA_3 group showed high accumulation in roots and other LEA proteins showed expression in embryos. Those results indicate an important role for abiotic stress. Zm00001d036454 contained a SNP associated with PRL in the Combined panel. It belongs to the Tetratricopeptide repeat superfamily proteins. Wei and Han (2017) investigated the tetratricopeptide repeat gene family for Arabidopsis, rice, poplar, and maize and found many genes associated with root and stem growth or functions, meristem growth, post-embryonic cell division, and differentiation of distal tissues in the root. The gene Zm00001d038809 contains a significant (*P*<3.83 × 10^−6^) SNP associated with LRL in the Combined panel. It belongs to the protein kinase superfamily. Fan *et al.* (2018) investigated the expression of protein kinases in maize, rice, and Arabidopsis and found genes with different functions in the plant, but a large number were highly expressed in maize roots. Our results suggest genes with possible influence on root traits that can be useful in breeding programs. However, these genes need to be validated under field conditions.

### Genomic prediction

In the last 15 years, genomic prediction has become an important tool to predict whether untested genotypes will increase breeding efficiency and the genetic gain in maize breeding programs (Crossa *et al.*, 2010; Edriss *et al.*, 2017). Researchers have used SNP markers to predict maize single crosses (Viana *et al.*, 2018), evaluate maize yield while considering genotype × environment interactions (Millet *et al.*, 2019), and combine SNP with different types of omics data to improve prediction accuracies (Xu *et al.*, 2017; Schrag *et al.*, 2018). Our study combined two maize association panels, BGEM and Ames, with different genetic backgrounds, which were genotyped with SNP markers and characterized for root traits at seedling stage (Fig. 4). This allowed us to assess prediction accuracies within, across, and in the combined populations or subpopulations for root traits in maize.

Several factors can affect the accuracy of genomic selection, such as training population size, genetic relationship, heritability, marker density, and statistical models (Liu *et al.*, 2015; Cerrudo *et al.*, 2018; Edwards *et al.*, 2019; Zhang *et al.*, 2019). In our study, genomic prediction by subpopulation showed lower accuracies; prediction within the original panels, Ames and BGEM, presented slightly better results than by subpopulation; and higher accuracies were found in the combined panels. In addition, we observed that genetic relationships between training and validation sets are more important than training population size. We found higher accuracies within SS Combined using 140 individuals as the training set compared with using the Ames panel with 379 individuals as the training set to predict BGEM performance.

Different statistical approaches have been used to determine breeding values from genomic prediction models (Crossa *et al.*, 2017; Alves *et al.*, 2019). Previous studies have reported better performance of the Bayesian approaches for genomic selection over other methods (Daetwyler *et al.*, 2010; Shikha *et al.*, 2017). The assumptions for the marker effects need to match the genetic architecture of target traits for the method be effective (Clark *et al.*, 2011; Zhao *et al.*, 2013). Bayesian models focus on QTL–marker associations, while RR-BLUP relies more on kinship (Habier *et al.*, 2007). Hence, if there are few QTLs with large effects, the Bayesian approaches will result in a better accuracy over RR-BLUP. Conversely, if there are many small-effect QTLs, the accuracies will be similar (Lin *et al.*, 2014). In our study, BayesC marginally outperformed the other methods.

The main goal of breeding is to select the best genotypes in early generations, saving time and money. GWAS and genomic prediction are powerful approaches that can be integrated in a breeding program to identify molecular markers associated to target traits and estimate GEBVs of individuals to save resources predicting and selecting untested genotypes. Recent progress in developing new DNA sequencing and marker technologies along with other -omics approaches will produce a large amount of data. Combining large datasets will be routine in breeding programs enabling geneticists to analyse data gathered from different experiments to identify biological causes for phenotypic variation in complex traits more reliably. As well, a standardized method for root phenotyping is important to expand this approach across the community and over time. However, statistical methods need to improve with computational speed to handle the continuously increasing sample size and marker density. In conclusion, combining results across studies is useful, even with different genetic backgrounds, to improve the prediction accuracy and detect polymorphisms associated with maize seedling root traits. One approach to handle these large datasets, as shown in this study, is to assign individuals to subpopulations by population structure analysis. The genes found can be further studied to help understand the genetic basis of root development and to manipulate root architecture.

## Supplementary data

The following supplementary data are available at *JXB* online.

Table S1. The 679 maize lines comprising the Combined panel, pedigree, breeding program, heterotic group, and subpopulation by fasStructure.

erac236_suppl_Supplementary_Table_1

## Data Availability

Genotypic data can be found openly available at http://cbsusrv04.tc.cornell.edu/users/panzea/download.aspx?filegroupid=4
