# Supplementary material for: Combining datasets for maize root seedling traits increases the power of GWAS and genomic prediction accuracies
Source: J Exp Bot. 2022 May 24;73(16):5460–73. doi: 10.1093/jxb/erac236 (PMC9467658; doi:10.1093/jxb/erac236)
Supplement: erac236_suppl_Supplementary_Table_1 [file erac236_suppl_supplementary_table_1.pdf]

Table S1. The 679 maize lines comprising the Combined panel, pedigree, breeding program, heterotic group and subpopulation by fasStructure.

Accession	Panel	pedigree	Breeding program	Heterotic Group	fastStructure
Ind_0001	BGEM	(ALTIPLANO BOV903/PHZ51)/PHZ51 #002-(2n)-003	Bolivia	non-stiff stalk	NSS BGEM
Ind_0002	BGEM	(ALTIPLANO BOV903/PHZ51)/PHZ51 #003-(2n)-001	Bolivia	non-stiff stalk	NSS BGEM
Ind_0003	BGEM	(ALTIPLANO BOV903/PHZ51)/PHZ51 #005-(2n)-001	Bolivia	non-stiff stalk	NSS BGEM
Ind_0004	BGEM	(ALTIPLANO BOV903/PHZ51)/PHZ51 #005-(2n)-003	Bolivia	non-stiff stalk	NSS BGEM
Ind_0005	BGEM	(ALTIPLANO BOV903/PHZ51)/PHZ51 #005-(2n)-005	Bolivia	non-stiff stalk	SSS BGEM
Ind_0033	BGEM	((Arequipeno - ARQ 1/PHZ51 B)/PHZ51)-(2n)-001	GEM	non-stiff stalk	NSS BGEM
Ind_0034	BGEM	((Arizona - LIB 16/PHZ51 B)/PHZ51)-(2n)-002	Peru	non-stiff stalk	NSS BGEM
Ind_0035	BGEM	((Arizona - LIB 16/PHZ51 B)/PHZ51)-(2n)-003	Peru	non-stiff stalk	NSS BGEM
Ind_0040	BGEM	(BOFO DGO123/PHZ51)/PHZ51 #002-(2n)-001	Mexico	non-stiff stalk	NSS BGEM
Ind_0041	BGEM	(BOFO DGO123/PHZ51)/PHZ51 #002-(2n)-002	Mexico	non-stiff stalk	NSS BGEM
Ind_0050	BGEM	(CANDELA ECU531/PHZ51)/PHZ51 #003-(2n)-001	Ecuador	non-stiff stalk	NSS BGEM
Ind_0053	BGEM	((Capio rosado - ARG 460/PHZ51)/PHZ51)-(2n)-001	Argentina	non-stiff stalk	NSS BGEM
Ind_0054	BGEM	(CHANDELLE CUB68 CI/PHZ51)/PHZ51 #004-(2n)-001	GEM	non-stiff stalk	NSS BGEM
Ind_0055	BGEM	(CHANDELLE CUB68 CI/PHZ51)/PHZ51 #006-(2n)-001	GEM	non-stiff stalk	NSS BGEM
Ind_0056	BGEM	(PHZ51/CON PN CUZ13)/PHZ51 #002-(2n)-001	Peru	non-stiff stalk	NSS BGEM
Ind_0057	BGEM	(PHZ51/CON PN CUZ13)/PHZ51 #007-(2n)-001	Peru	non-stiff stalk	NSS BGEM
Ind_0075	BGEM	(CUZCO CUZ217/PHZ51)/PHZ51 #002-(2n)-001	GEM	non-stiff stalk	SSS Ames
Ind_0076	BGEM	(CUZCO CUZ217/PHZ51)/PHZ51 #004-(2n)-001	GEM	non-stiff stalk	NSS BGEM
Ind_0077	BGEM	(DULCILLO DEL NO SON57/PHZ51)/PHZ51 #001-(2n)-001	Mexico	non-stiff stalk	NSS BGEM
Ind_0078	BGEM	(DULCILLO DEL NO SON57/PHZ51)/PHZ51 #002-(2n)-002	Mexico	non-stiff stalk	NSS BGEM
Ind_0079	BGEM	(DULCILLO DEL NO SON57/PHZ51)/PHZ51 #002-(2n)-003	Mexico	non-stiff stalk	NSS BGEM
Ind_0080	BGEM	(DULCILLO DEL NO SON57/PHZ51)/PHZ51 #002-(2n)-004	Mexico	non-stiff stalk	NSS BGEM
Ind_0081	BGEM	(DULCILLO DEL NO SON57/PHZ51)/PHZ51 #003-(2n)-002	Mexico	non-stiff stalk	NSS BGEM
Ind_0086	BGEM	(EARLY CARIBBEAN MAR9/PHZ51)/PHZ51 #006-(2n)-003	Martinique	non-stiff stalk	NSSS Ames
Ind_0092	BGEM	(ELOTES OCCIDENT NAY29/PHZ51)/PHZ51 #002-(2n)-001	Mexico	non-stiff stalk	NSS BGEM
Ind_0093	BGEM	(ELOTES OCCIDENT NAY29/PHZ51)/PHZ51 #004-(2n)-002	Mexico	non-stiff stalk	NSS BGEM
Ind_0094	BGEM	(ELOTES OCCIDENT NAY29/PHZ51)/PHZ51 #004-(2n)-003	Mexico	non-stiff stalk	NSS BGEM
Ind_0095	BGEM	(ELOTES OCCIDENT NAY29/PHZ51)/PHZ51 #004-(2n)-004	Mexico	non-stiff stalk	SSS BGEM
Ind_0097	BGEM	(ELOTES OCCIDENT DGO236/PHZ51)/PHZ51 #004-(2n)-002	Mexico	non-stiff stalk	NSS BGEM
Ind_0098	BGEM	(ELOTES OCCIDENT DGO236/PHZ51)/PHZ51 #007-(2n)-001	Mexico	non-stiff stalk	NSS BGEM
Ind_0107	BGEM	((Jora - ANC 1/PHZ51 B)/PHZ51)-(2n)-001	Peru	non-stiff stalk	NSS BGEM
Ind_0108	BGEM	((Jora - ANC 1/PHZ51 B)/PHZ51)-(2n)-002	Peru	non-stiff stalk	NSSS Ames
Ind_0109	BGEM	((Jora - ANC 1/PHZ51 B)/PHZ51)-(2n)-003	Peru	non-stiff stalk	NSS BGEM
Ind_0110	BGEM	(KARAPAMPA BOV978/PHZ51)/PHZ51 #003-(2n)-001	Bolivia	non-stiff stalk	NSS BGEM
Ind_0111	BGEM	(KARAPAMPA BOV978/PHZ51)/PHZ51 #003-(2n)-002	Bolivia	non-stiff stalk	NSS BGEM
Ind_0112	BGEM	(KARAPAMPA BOV978/PHZ51)/PHZ51 #003-(2n)-003	Bolivia	non-stiff stalk	NSS BGEM
Ind_0113	BGEM	(KARAPAMPA BOV978/PHZ51)/PHZ51 #003-(2n)-004	Bolivia	non-stiff stalk	NSS BGEM
Ind_0114	BGEM	(KARAPAMPA BOV978/PHZ51)/PHZ51 #003-(2n)-006	Bolivia	non-stiff stalk	SSS BGEM
Ind_0115	BGEM	(KARAPAMPA BOV978/PHZ51)/PHZ51 #003-(2n)-007	Bolivia	non-stiff stalk	NSS BGEM
Ind_0116	BGEM	(KARAPAMPA BOV978/PHZ51)/PHZ51 #003-(2n)-008	Bolivia	non-stiff stalk	NSS BGEM
Ind_0117	BGEM	(KARAPAMPA BOV978/PHZ51)/PHZ51 #004-(2n)-005	Bolivia	non-stiff stalk	SSS BGEM
Ind_0123	BGEM	(PHZ51/KCELO BOV948)/PHZ51 #001-(2n)-001	Bolivia	non-stiff stalk	NSS BGEM
Ind_0124	BGEM	(PHZ51/KCELO BOV948)/PHZ51 #001-(2n)-002	Bolivia	non-stiff stalk	NSS BGEM
Ind_0125	BGEM	(PHZ51/KCELO BOV948)/PHZ51 #001-(2n)-005	Bolivia	non-stiff stalk	NSS BGEM
Ind_0126	BGEM	(PHZ51/KCELO BOV948)/PHZ51 #003-(2n)-001	Bolivia	non-stiff stalk	NSS BGEM
Ind_0127	BGEM	(PHZ51/KCELO BOV948)/PHZ51 #003-(2n)-002	Bolivia	non-stiff stalk	NSS BGEM
Ind_0128	BGEM	(PHZ51/KCELO BOV948)/PHZ51 #004-(2n)-001	Bolivia	non-stiff stalk	NSS BGEM
Ind_0129	BGEM	(PHZ51/KCELO BOV948)/PHZ51 #004-(2n)-002	Bolivia	non-stiff stalk	NSS BGEM
Ind_0134	BGEM	(PHZ51/MISHC ECU321)/PHZ51 #004-(2n)-002	Ecuador	non-stiff stalk	NSS BGEM
Ind_0135	BGEM	(PHZ51/MISHC ECU321)/PHZ51 #004-(2n)-003	Ecuador	non-stiff stalk	NSS BGEM
Ind_0139	BGEM	CUBA164:N(PHZ51)/(PHZ51)-(2n)-002	Cuba	non-stiff stalk	NSS BGEM
Ind_0155	BGEM	(PHZ51/(PHZ51/MOROCHO APC67) #001-(2n))-001	Peru	non-stiff stalk	NSS BGEM
Ind_0159	BGEM	(ONAVENO SON24/PHZ51)/PHZ51 #001-(2n)-002	Mexico	non-stiff stalk	NSS BGEM
Ind_0160	BGEM	(ONAVENO SON24/PHZ51)/PHZ51 #004-(2n)-001	Mexico	non-stiff stalk	NSS BGEM
Ind_0161	BGEM	(ONAVENO SON24/PHZ51)/PHZ51 #005-(2n)-001	Mexico	non-stiff stalk	NSS BGEM
Ind_0162	BGEM	(ONAVENO SON24/PHZ51)/PHZ51 #005-(2n)-002	Mexico	non-stiff stalk	NSS BGEM
Ind_0163	BGEM	(ONAVENO SON24/PHZ51)/PHZ51 #005-(2n)-003	Mexico	non-stiff stalk	NSS BGEM
Ind_0168	BGEM	((Patillo - ECU 417/PHZ51)/PHZ51)-(2n)-002	Ecuador	non-stiff stalk	NSS BGEM
Ind_0169	BGEM	(PATILLO BOV493/PHZ51)/PHZ51 #001-(2n)-001	Bolivia	non-stiff stalk	NSS BGEM
Ind_0170	BGEM	(PATILLO BOV493/PHZ51)/PHZ51 #003-(2n)-001	Bolivia	non-stiff stalk	NSS BGEM
Ind_0171	BGEM	(PATILLO BOV493/PHZ51)/PHZ51 #003-(2n)-002	Bolivia	non-stiff stalk	NSS BGEM
Ind_0172	BGEM	(PATILLO GRANDE BOV649/PHZ51)/PHZ51 #005-(2n)-001	Bolivia	non-stiff stalk	NSS BGEM
Ind_0173	BGEM	(PATILLO GRANDE BOV649/PHZ51)/PHZ51 #006-(2n)-003	Bolivia	non-stiff stalk	NSS BGEM
Ind_0177	BGEM	(PIRA TOL405/PHZ51)/PHZ51 #001-(2n)-001	Colombia	non-stiff stalk	NSS BGEM
Ind_0178	BGEM	(PIRA TOL405/PHZ51)/PHZ51 #002-(2n)-001	Colombia	non-stiff stalk	NSS BGEM
Ind_0179	BGEM	(PHZ51/PISAN BOV344)/PHZ51 #001-(2n)-002	Bolivia	non-stiff stalk	NSS BGEM
Ind_0180	BGEM	(PHZ51/PISAN BOV344)/PHZ51 #001-(2n)-003	Bolivia	non-stiff stalk	NSS BGEM
Ind_0181	BGEM	(PHZ51/PISAN BOV344)/PHZ51 #001-(2n)-004	Bolivia	non-stiff stalk	NSS BGEM
Ind_0182	BGEM	(PHZ51/PISAN BOV344)/PHZ51 #001-(2n)-005	Bolivia	non-stiff stalk	NSS BGEM
Ind_0183	BGEM	(PHZ51/PISAN BOV344)/PHZ51 #001-(2n)-006	Bolivia	non-stiff stalk	NSS BGEM
Ind_0184	BGEM	(PHZ51/PISAN BOV344)/PHZ51 #002-(2n)-001	Bolivia	non-stiff stalk	NSS BGEM
Ind_0185	BGEM	(PHZ51/PISAN BOV344)/PHZ51 #002-(2n)-002	Bolivia	non-stiff stalk	NSS BGEM

Table S1. The 679 maize lines comprising the Combined panel, pedigree, breeding program, heterotic group and subpopulation by fasStructure.

**Accession**	**Panel**	**pedigree**	**Breeding program**	**Heterotic Group**	**fastStructure**
Ind_0186	BGEM	(PHZ51/PISAN BOV344)/PHZ51 #003-(2n)-001	Bolivia	non-stiff stalk	NSS BGEM
Ind_0187	BGEM	(PHZ51/PISAN BOV344)/PHZ51 #003-(2n)-002	Bolivia	non-stiff stalk	NSS BGEM
Ind_0190	BGEM	(POJ CHICO BOV800/PHZ51)/PHZ51 #001-(2n)-001	Bolivia	non-stiff stalk	SSS BGEM
Ind_0191	BGEM	(POJ CHICO BOV800/PHZ51)/PHZ51 #006-(2n)-001	Bolivia	non-stiff stalk	NSS BGEM
Ind_0198	BGEM	(SG HUANCAV JUN164/PHZ51)/PHZ51 #005-(2n)-001	GEM	non-stiff stalk	NSS BGEM
Ind_0199	BGEM	(SG HUANCAV JUN164/PHZ51)/PHZ51 #005-(2n)-002	GEM	non-stiff stalk	NSS BGEM
Ind_0200	BGEM	(SG HUANCAV JUN164/PHZ51)/PHZ51 #005-(2n)-003	GEM	non-stiff stalk	NSS BGEM
Ind_0202	BGEM	BR105:N(PHZ51)/(PHZ51)-(2n)-001	GEM	non-stiff stalk	SSS BGEM
Ind_0203	BGEM	BR105:N(PHZ51)/(PHZ51)-(2n)-003	GEM	non-stiff stalk	NSS BGEM
Ind_0204	BGEM	BR105:N(PHZ51)/(PHZ51)-(2n)-004	GEM	non-stiff stalk	NSS BGEM
Ind_0205	BGEM	BR105:N(PHZ51)/(PHZ51)-(2n)-005	GEM	non-stiff stalk	NSS BGEM
Ind_0206	BGEM	BR105:N(PHZ51)/(PHZ51)-(2n)-007	GEM	non-stiff stalk	NSS BGEM
Ind_0207	BGEM	((Tehua - CHS29/PHZ51 B)/PHZ51)-(2n)-004	GEM	non-stiff stalk	SSS BGEM
Ind_0208	BGEM	BR106:N(PHZ51)/(PHZ51)-(2n)-001	GEM	non-stiff stalk	NSS BGEM
Ind_0209	BGEM	((Tuxpeño - GUA 456/PHZ51 B)/PHZ51)-(2n)-002	GEM	non-stiff stalk	NSS BGEM
Ind_0211	BGEM	(YUCATAN TOL389 ICA/PHZ51)/PHZ51 #003-(2n)-001	GEM	non-stiff stalk	NSS BGEM
Ind_0212	BGEM	(YUCATAN TOL389 ICA/PHZ51)/PHZ51 #003-(2n)-003	GEM	non-stiff stalk	NSS BGEM
Ind_0213	BGEM	(YUCATAN TOL389 ICA/PHZ51)/PHZ51 #005-(2n)-001	GEM	non-stiff stalk	NSS BGEM
Ind_0214	BGEM	(YUCATAN TOL389 ICA/PHZ51)/PHZ51 #005-(2n)-002	GEM	non-stiff stalk	NSS BGEM
Ind_0215	BGEM	(YUCATAN TOL389 ICA/PHZ51)/PHZ51 #005-(2n)-003	GEM	non-stiff stalk	NSS BGEM
Ind_0216	BGEM	(YUCATAN TOL389 ICA/PHZ51)/PHZ51 #005-(2n)-005	GEM	non-stiff stalk	NSS BGEM
Ind_0220	BGEM	(YUNGUENO BOV362/PHZ51)/PHZ51 #004-(2n)-001	GEM	non-stiff stalk	NSS BGEM
Ind_0221	BGEM	(YUNGUENO BOV362/PHZ51)/PHZ51 #005-(2n)-002	GEM	non-stiff stalk	NSS BGEM
Ind_0222	BGEM	(YUNGUENO BOV362/PHZ51)/PHZ51 #006-(2n)-002	GEM	non-stiff stalk	SSS BGEM
Ind_0223	BGEM	(YUNGUENO BOV362/PHZ51)/PHZ51 #006-(2n)-004	GEM	non-stiff stalk	NSS BGEM
Ind_0227	BGEM	((Avati Moroti Guapi - PAG 139/PHZ51 B)/PHZ51)-(2n)-001	Paraguay	non-stiff stalk	NSS BGEM
Ind_0230	BGEM	((Chirimito - VEN 703/PHZ51 B)/PHZ51)-(2n)-001	GEM	non-stiff stalk	NSS BGEM
Ind_0233	BGEM	((PHZ51/Jala - JAL 44)/PHZ51)-(2n)-001	GEM	non-stiff stalk	NSS BGEM
Ind_0234	BGEM	((Puya - MAG 355/PHZ51 B)/PHZ51)-(2n)-001	GEM	non-stiff stalk	SSS BGEM
Ind_0242	BGEM	((Amarillo Huancabamba - PIU 38B/PHZ51 B)/PHZ51)-(2n)-001	GEM	non-stiff stalk	NSS BGEM
Ind_0244	BGEM	((ARAGUITO - VEN678/PHZ51)/PHZ51)-(2n)-001	GEM	non-stiff stalk	NSS BGEM
Ind_0245	BGEM	((Avati Moroti Ti - PAG 124/PHZ51)/PHZ51)-(2n)-001	Paraguay	non-stiff stalk	NSS BGEM
Ind_0248	BGEM	((Canilla - VEN693/PHZ51 B)/PHZ51)-(2n)-001	GEM	non-stiff stalk	NSS BGEM
Ind_0249	BGEM	((Caraja - 1692 USDA/PHZ51 B)/PHZ51)-(2n)-001	GEM	non-stiff stalk	NSS BGEM
Ind_0252	BGEM	(PHZ51/CON PN CUZ13)/PHZ51 #004-(2n)-001	GEM	non-stiff stalk	NSS BGEM
Ind_0256	BGEM	((Cubano dentado - BOV 585/PHZ51)/PHZ51)-(2n)-002	GEM	non-stiff stalk	NSS BGEM
Ind_0257	BGEM	(CURAGUA GRANDE CHI303/PHZ51)/PHZ51 #005-(2n)-001	GEM	non-stiff stalk	NSS BGEM
Ind_0258	BGEM	(CUZCO CUZ217/PHZ51)/PHZ51 #002-(2n)-002	GEM	non-stiff stalk	SSS BGEM
Ind_0261	BGEM	(EARLY CARIBBEAN MAR9/PHZ51)/PHZ51 #002-(2n)-002	GEM	non-stiff stalk	NSS BGEM
Ind_0262	BGEM	(EARLY CARIBBEAN MAR9/PHZ51)/PHZ51 #003-(2n)-002	GEM	non-stiff stalk	NSS BGEM
Ind_0263	BGEM	(EARLY CARIBBEAN MAR9/PHZ51)/PHZ51 #006-(2n)-001	GEM	non-stiff stalk	NSS BGEM
Ind_0264	BGEM	(EARLY CARIBBEAN MAR9/PHZ51)/PHZ51 #006-(2n)-002	GEM	non-stiff stalk	NSS BGEM
Ind_0268	BGEM	(KARAPAMPA BOV978/PHZ51)/PHZ51 #003-(2n)-005	GEM	non-stiff stalk	NSS BGEM
Ind_0269	BGEM	(KARAPAMPA BOV978/PHZ51)/PHZ51 #003-(2n)-009	GEM	non-stiff stalk	NSS BGEM
Ind_0270	BGEM	(PHZ51/MISHC ECU321)/PHZ51 #004-(2n)-004	GEM	non-stiff stalk	NSS BGEM
Ind_0275	BGEM	(MORADO CANTENO LIM55/PHZ51)/PHZ51 #005-(2n)-001	GEM	non-stiff stalk	NSS BGEM
Ind_0278	BGEM	(ONAVENO SON24/PHZ51)/PHZ51 #001-(2n)-001	GEM	non-stiff stalk	NSS BGEM
Ind_0279	BGEM	((Patillo - ECU 417/PHZ51)/PHZ51)-(2n)-001	GEM	non-stiff stalk	NSS BGEM
Ind_0280	BGEM	(PIRA TOL405/PHZ51)/PHZ51 #003-(2n)-001	GEM	non-stiff stalk	SSS Ames
Ind_0282	BGEM	(POJ CHICO BOV800/PHZ51)/PHZ51 #004-(2n)-001	GEM	non-stiff stalk	NSS BGEM
Ind_0289	BGEM	((Reventador - NAY 39/PHZ51)/PHZ51)-(2n)-001	GEM	non-stiff stalk	NSS BGEM
Ind_0292	BGEM	((Semi dentado paulista - PAG I/PHZ51 B)/PHZ51)-(2n)-002	GEM	non-stiff stalk	NSS BGEM
Ind_0293	BGEM	((Tehua - CHS29/PHZ51 B)/PHZ51)-(2n)-002	GEM	non-stiff stalk	NSS BGEM
Ind_0296	BGEM	((Tuxpeño - GUA 456/PHZ51 B)/PHZ51)-(2n)-001	GEM	non-stiff stalk	NSS BGEM
Ind_0297	BGEM	((Vandeño - GRO 96/PHZ51 B)/PHZ51)-(2n)-001	GEM	non-stiff stalk	NSS BGEM
Ind_0298	BGEM	(YUNGUENO BOV362/PHZ51)/PHZ51 #005-(2n)-001	GEM	non-stiff stalk	NSS BGEM
Ind_0299	BGEM	(YUNGUENO BOV362/PHZ51)/PHZ51 #006-(2n)-001	GEM	non-stiff stalk	NSS BGEM
Ind_0300	BGEM	(YUNGUENO BOV362/PHZ51)/PHZ51 #006-(2n)-003	GEM	non-stiff stalk	NSS BGEM
Ind_0302	BGEM	PHZ51	GEM	non-stiff stalk	NSS BGEM
Ind_0006	BGEM	(ALTIPLANO BOV903/PHB47)/PHB47 #001-(2n)-002	Bolivia	stiff stalk	SSS BGEM
Ind_0007	BGEM	(ALTIPLANO BOV903/PHB47)/PHB47 #001-(2n)-004	Bolivia	stiff stalk	NSS BGEM
Ind_0008	BGEM	(ALTIPLANO BOV903/PHB47)/PHB47 #001-(2n)-005	Bolivia	stiff stalk	SSS BGEM
Ind_0009	BGEM	(ALTIPLANO BOV903/PHB47)/PHB47 #003-(2n)-002	Bolivia	stiff stalk	SSS BGEM
Ind_0010	BGEM	(ALTIPLANO BOV903/PHB47)/PHB47 #004-(2n)-002	Bolivia	stiff stalk	SSS BGEM
Ind_0011	BGEM	(ALTIPLANO BOV903/PHB47)/PHB47 #004-(2n)-003	Bolivia	stiff stalk	SSS BGEM
Ind_0012	BGEM	(ALTIPLANO BOV903/PHB47)/PHB47 #005-(2n)-001	Bolivia	stiff stalk	SSS BGEM
Ind_0013	BGEM	(ANDAQUI CAQ307 FT/PHB47)/PHB47 #003-(2n)-001	Colombia	stiff stalk	SSS BGEM
Ind_0014	BGEM	(ANDAQUI CAQ307 FT/PHB47)/PHB47 #003-(2n)-002	Colombia	stiff stalk	SSS BGEM
Ind_0015	BGEM	(ANDAQUI CAQ307 FT/PHB47)/PHB47 #003-(2n)-004	Colombia	stiff stalk	SSS BGEM
Ind_0016	BGEM	(ANDAQUI CAQ307 FT/PHB47)/PHB47 #003-(2n)-006	Colombia	stiff stalk	SSS BGEM
Ind_0017	BGEM	(ANDAQUI CAQ307 FT/PHB47)/PHB47 #003-(2n)-007	Colombia	stiff stalk	SSS BGEM
Ind_0018	BGEM	(ANDAQUI CAQ307 FT/PHB47)/PHB47 #005-(2n)-001	Colombia	stiff stalk	SSS BGEM

Table S1. The 679 maize lines comprising the Combined panel, pedigree, breeding program, heterotic group and subpopulation by fasStructure.

Accession	Panel	pedigree	Breeding program	Heterotic Group	fastStructure
Ind_0019	BGEM	(ANDAQUI CAQ307 FT/PHB47)/PHB47 #006-(2n)-001	Colombia	stiff stalk	SSS BGEM
Ind_0020	BGEM	(ANDAQUI CAQ307 FT/PHB47)/PHB47 #007-(2n)-002	Colombia	stiff stalk	SSS BGEM
Ind_0021	BGEM	(ARAGUITO VEN678/PHB47)/PHB47 #003-(2n)-002	Venezuela	stiff stalk	SSS BGEM
Ind_0022	BGEM	(ARAGUITO VEN678/PHB47)/PHB47 #003-(2n)-003	Venezuela	stiff stalk	SSS BGEM
Ind_0023	BGEM	(ARAGUITO VEN678/PHB47)/PHB47 #003-(2n)-004	Venezuela	stiff stalk	SSS BGEM
Ind_0024	BGEM	(ARAGUITO VEN678/PHB47)/PHB47 #005-(2n)-003	Venezuela	stiff stalk	SSS BGEM
Ind_0025	BGEM	(ARAGUITO VEN678/PHB47)/PHB47 #005-(2n)-004	Venezuela	stiff stalk	SSS BGEM
Ind_0026	BGEM	(ARAGUITO VEN678/PHB47)/PHB47 #005-(2n)-005	Venezuela	stiff stalk	SSS BGEM
Ind_0027	BGEM	(ARAGUITO VEN678/PHB47)/PHB47 #006-(2n)-002	Venezuela	stiff stalk	SSS BGEM
Ind_0028	BGEM	(ARAGUITO VEN678/PHB47)/PHB47 #006-(2n)-004	Venezuela	stiff stalk	SSS BGEM
Ind_0029	BGEM	(ARAGUITO VEN678/PHB47)/PHB47 #006-(2n)-006	Venezuela	stiff stalk	SSS BGEM
Ind_0030	BGEM	(ARAGUITO VEN678/PHB47)/PHB47 #006-(2n)-007	Venezuela	stiff stalk	SSS BGEM
Ind_0031	BGEM	(ARAGUITO VEN678/PHB47)/PHB47 #006-(2n)-008	Venezuela	stiff stalk	SSS BGEM
Ind_0032	BGEM	(ARAGUITO VEN678/PHB47)/PHB47 #006-(2n)-009	Venezuela	stiff stalk	SSS BGEM
Ind_0036	BGEM	((Arizona - LIB 16/PHB47 B)/PHB47)-(2n)-001	Peru	stiff stalk	SSS BGEM
Ind_0037	BGEM	((Avati Moroti Guapi - PAG 139/PHB47 B)/PHB47)-(2n)-003	Paraguay	stiff stalk	SSS BGEM
Ind_0038	BGEM	((Avati Moroti Guapi - PAG 139/PHB47 B)/PHB47)-(2n)-004	Paraguay	stiff stalk	SSS BGEM
Ind_0039	BGEM	((Blanco Blandito - ECU 523/PHB47 B)/PHB47)-(2n)-001	Ecuador	stiff stalk	SSS BGEM
Ind_0042	BGEM	(BOFO DGO123/PHB47)/PHB47 #001-(2n)-001	Mexico	stiff stalk	SSS BGEM
Ind_0043	BGEM	(BOFO DGO123/PHB47)/PHB47 #002-(2n)-003	Mexico	stiff stalk	SSS BGEM
Ind_0044	BGEM	(BOFO DGO123/PHB47)/PHB47 #003-(2n)-003	Mexico	stiff stalk	SSS BGEM
Ind_0045	BGEM	(BOFO DGO123/PHB47)/PHB47 #004-(2n)-001	Mexico	stiff stalk	SSS BGEM
Ind_0046	BGEM	(BOFO DGO123/PHB47)/PHB47 #004-(2n)-002	Mexico	stiff stalk	SSS BGEM
Ind_0047	BGEM	(BOFO DGO123/PHB47)/PHB47 #004-(2n)-003	Mexico	stiff stalk	NSS BGEM
Ind_0048	BGEM	(CABUYA SAN316 FTC/PHB47)/PHB47 #006-(2n)-001	GEM	stiff stalk	SSS BGEM
Ind_0049	BGEM	(CAMELIA CHI411/PHB47)/PHB47 #005-(2n)-002	Chile	stiff stalk	SSS BGEM
Ind_0051	BGEM	(CANDELA ECU531/PHB47)/PHB47 #003-(2n)-001	Ecuador	stiff stalk	SSS BGEM
Ind_0052	BGEM	(CANDELA ECU531/PHB47)/PHB47 #003-(2n)-002	Ecuador	stiff stalk	SSS BGEM
Ind_0058	BGEM	(PHB47/CON PUNT CUZ13)/PHB47 #002-(2n)-001	Peru	stiff stalk	SSS BGEM
Ind_0059	BGEM	(PHB47/CON PUNT CUZ13)/PHB47 #005-(2n)-001	Peru	stiff stalk	SSS BGEM
Ind_0060	BGEM	(CON NORT ZAC161/PHB47)/PHB47 #002-(2n)-002	Mexico	stiff stalk	SSS BGEM
Ind_0061	BGEM	(CON NORT ZAC161/PHB47)/PHB47 #003-(2n)-001	Mexico	stiff stalk	SSS BGEM
Ind_0062	BGEM	(CON NORT ZAC161/PHB47)/PHB47 #004-(2n)-001	Mexico	stiff stalk	SSS BGEM
Ind_0063	BGEM	(CON NORT ZAC161/PHB47)/PHB47 #004-(2n)-004	Mexico	stiff stalk	SSS BGEM
Ind_0064	BGEM	(CON NORT ZAC161/PHB47)/PHB47 #005-(2n)-001	Mexico	stiff stalk	SSS BGEM
Ind_0065	BGEM	(CON NORT ZAC161/PHB47)/PHB47 #005-(2n)-003	Mexico	stiff stalk	SSS BGEM
Ind_0066	BGEM	(CON NORT ZAC161/PHB47)/PHB47 #005-(2n)-005	Mexico	stiff stalk	SSS BGEM
Ind_0067	BGEM	(CON NORT ZAC161/PHB47)/PHB47 #005-(2n)-006	Mexico	stiff stalk	NSSS Ames
Ind_0068	BGEM	(CRISTALINO AMAR AR21004/PHB47)/PHB47 #001-(2n)-002	Argentina	stiff stalk	NSS BGEM
Ind_0069	BGEM	(CRISTALINO AMAR AR21004/PHB47)/PHB47 #005-(2n)-003	Argentina	stiff stalk	SSS BGEM
Ind_0070	BGEM	(CRISTALINO AMAR AR21004/PHB47)/PHB47 #005-(2n)-004	Argentina	stiff stalk	SSS BGEM
Ind_0071	BGEM	(CRISTALINO AMAR AR21004/PHB47)/PHB47 #007-(2n)-001	Argentina	stiff stalk	SSS BGEM
Ind_0072	BGEM	(CRISTALINO AMAR AR21004/PHB47)/PHB47 #007-(2n)-002	Argentina	stiff stalk	SSS BGEM
Ind_0073	BGEM	((Cubano dentado - BOV 585/PHB47 B)/PHB47)-(2n)-001	Bolivia	stiff stalk	SSS BGEM
Ind_0074	BGEM	(CURAGUA GRANDE CHI303/PHB47)/PHB47 #004-(2n)-001	GEM	stiff stalk	SSS BGEM
Ind_0082	BGEM	(DULCILLO DE NO SON57/PHB47)/PHB47 #002-(2n)-001	Mexico	stiff stalk	SSS BGEM
Ind_0083	BGEM	(DULCILLO DE NO SON57/PHB47)/PHB47 #002-(2n)-002	Mexico	stiff stalk	SSS BGEM
Ind_0084	BGEM	(DULCILLO DE NO SON57/PHB47)/PHB47 #005-(2n)-003	Mexico	stiff stalk	NSS BGEM
Ind_0085	BGEM	(DULCILLO DE NO SON57/PHB47)/PHB47 #006-(2n)-001	Mexico	stiff stalk	SSS BGEM
Ind_0087	BGEM	(EARLY CARIBBEAN MAR9/PHB47)/PHB47 #001-(2n)-002	Martinique	stiff stalk	SSS BGEM
Ind_0088	BGEM	(EARLY CARIBBEAN MAR9/PHB47)/PHB47 #006-(2n)-001	Martinique	stiff stalk	SSS BGEM
Ind_0089	BGEM	(EARLY CARIBBEAN MAR9/PHB47)/PHB47 #006-(2n)-002	Martinique	stiff stalk	SSS BGEM
Ind_0090	BGEM	(EARLY CARIBBEAN MAR9/PHB47)/PHB47 #007-(2n)-001	Martinique	stiff stalk	SSS BGEM
Ind_0091	BGEM	(EARLY CARIBBEAN MAR9/PHB47)/PHB47 #007-(2n)-005	Martinique	stiff stalk	SSS BGEM
Ind_0096	BGEM	(ELOTES OCCIDENT NAY29/PHB47)/PHB47 #002-(2n)-001	Mexico	stiff stalk	SSS BGEM
Ind_0099	BGEM	(PHB47/GORDO [CHH131]{CIMYT})-B-B-SIB-011-001-001-(2n)-001	Mexico	stiff stalk	SSS BGEM
Ind_0100	BGEM	(PHB47/GORDO [CHH131]{CIMYT})-B-B-SIB-011-001-001-(2n)-002	Mexico	stiff stalk	SSS BGEM
Ind_0101	BGEM	(PHB47/GORDO [CHH131]{CIMYT})-B-B-SIB-011-001-001-(2n)-003	Mexico	stiff stalk	SSS BGEM
Ind_0102	BGEM	(PHB47/GORDO [CHH131]{CIMYT})-B-B-SIB-011-001-001-(2n)-004	Mexico	stiff stalk	SSS BGEM
Ind_0103	BGEM	(PHB47/GORDO [CHH131]{CIMYT})-B-B-SIB-011-001-001-(2n)-005	Mexico	stiff stalk	SSS BGEM
Ind_0104	BGEM	(PHB47/GORDO [CHH131]{CIMYT})-B-B-SIB-011-001-001-(2n)-006	Mexico	stiff stalk	SSS BGEM
Ind_0105	BGEM	(PHB47/GORDO [CHH131]{CIMYT})-B-B-SIB-011-001-001-(2n)-007	Mexico	stiff stalk	SSS BGEM
Ind_0106	BGEM	(PHB47/GORDO [CHH131]{CIMYT})-B-B-SIB-019-001-001-(2n)-001	Mexico	stiff stalk	SSS BGEM
Ind_0118	BGEM	(KARAPAMPA BOV978/PHB47)/PHB47 #003-(2n)-001	Bolivia	stiff stalk	SSS BGEM
Ind_0119	BGEM	(KARAPAMPA BOV978/PHB47)/PHB47 #004-(2n)-001	Bolivia	stiff stalk	SSS BGEM
Ind_0120	BGEM	(KARAPAMPA BOV978/PHB47)/PHB47 #005-(2n)-002	Bolivia	stiff stalk	SSS BGEM
Ind_0121	BGEM	(KARAPAMPA BOV978/PHB47)/PHB47 #005-(2n)-003	Bolivia	stiff stalk	SSS BGEM
Ind_0122	BGEM	(KARAPAMPA BOV978/PHB47)/PHB47 #005-(2n)-005	Bolivia	stiff stalk	SSS BGEM
Ind_0130	BGEM	(PHB47/KCELO BOV948)/PHB47 #001-(2n)-001	Bolivia	stiff stalk	SSS BGEM
Ind_0131	BGEM	(PHB47/KCELO BOV948)/PHB47 #001-(2n)-002	Bolivia	stiff stalk	SSS BGEM
Ind_0132	BGEM	(PHB47/KCELO BOV948)/PHB47 #001-(2n)-003	Bolivia	stiff stalk	SSS BGEM
Ind_0133	BGEM	(PHB47/KCELO BOV948)/PHB47 #003-(2n)-002	Bolivia	stiff stalk	SSS BGEM

Table S1. The 679 maize lines comprising the Combined panel, pedigree, breeding program, heterotic group and subpopulation by fasStructure.

Accession	Panel	pedigree	Breeding program	Heterotic Group	fastStructure
Ind_0136	BGEM	(PHB47/MISHCA ECU321)/PHB47 #002-(2n)-001	Ecuador	stiff stalk	SSS BGEM
Ind_0137	BGEM	(PHB47/MISHCA ECU321)/PHB47 #003-(2n)-001	Ecuador	stiff stalk	SSS BGEM
Ind_0138	BGEM	(PHB47/MISHCA ECU321)/PHB47 #004-(2n)-001	Ecuador	stiff stalk	SSS BGEM
Ind_0140	BGEM	(MONTANA NAR625/PHB47)/PHB47 #003-(2n)-002	Colombia	stiff stalk	SSS BGEM
Ind_0141	BGEM	(MONTANA NAR625/PHB47)/PHB47 #003-(2n)-003	Colombia	stiff stalk	SSS BGEM
Ind_0142	BGEM	(MONTANA NAR625/PHB47)/PHB47 #006-(2n)-001	Colombia	stiff stalk	SSS BGEM
Ind_0143	BGEM	(MONTANA NAR625/PHB47)/PHB47 #006-(2n)-003	Colombia	stiff stalk	SSS BGEM
Ind_0144	BGEM	(MORADO BOV567/PHB47)/PHB47 #002-(2n)-001	Bolivia	stiff stalk	SSS BGEM
Ind_0145	BGEM	(MORADO BOV567/PHB47)/PHB47 #003-(2n)-001	Bolivia	stiff stalk	SSS BGEM
Ind_0146	BGEM	(MORADO BOV567/PHB47)/PHB47 #003-(2n)-002	Bolivia	stiff stalk	SSS BGEM
Ind_0147	BGEM	(MORADO BOV567/PHB47)/PHB47 #003-(2n)-003	Bolivia	stiff stalk	NSS BGEM
Ind_0148	BGEM	(MORADO BOV567/PHB47)/PHB47 #005-(2n)-002	Bolivia	stiff stalk	NSS BGEM
Ind_0149	BGEM	(MORADO BOV567/PHB47)/PHB47 #005-(2n)-003	Bolivia	stiff stalk	SSS BGEM
Ind_0150	BGEM	(MORADO BOV567/PHB47)/PHB47 #006-(2n)-002	Bolivia	stiff stalk	SSS BGEM
Ind_0151	BGEM	(MORADO BOV567/PHB47)/PHB47 #006-(2n)-003	Bolivia	stiff stalk	SSS BGEM
Ind_0152	BGEM	(MORADO CANTENO LIM55/PHB47)/PHB47 #001-(2n)-001	Peru	stiff stalk	NSSS Ames
Ind_0153	BGEM	(MORADO CANTENO LIM55/PHB47)/PHB47 #002-(2n)-002	Peru	stiff stalk	SSS BGEM
Ind_0154	BGEM	(MORADO CANTENO LIM55/PHB47)/PHB47 #002-(2n)-004	Peru	stiff stalk	SSS BGEM
Ind_0156	BGEM	(MOROCHO APUC77/PHB47)/PHB47 #003-(2n)-001	Peru	stiff stalk	NSS BGEM
Ind_0157	BGEM	(PHB47/MOROC APC67)/PHB47 #002-(2n)-001	Peru	stiff stalk	SSS BGEM
Ind_0158	BGEM	(PHB47/MOROC APC67)/PHB47 #006-(2n)-001	Peru	stiff stalk	SSS BGEM
Ind_0164	BGEM	(ONAVENO SON24/PHB47)/PHB47 #002-(2n)-001	Mexico	stiff stalk	SSS BGEM
Ind_0165	BGEM	(ONAVENO SON24/PHB47)/PHB47 #003-(2n)-001	Mexico	stiff stalk	SSS BGEM
Ind_0166	BGEM	(ONAVENO SON24/PHB47)/PHB47 #004-(2n)-001	Mexico	stiff stalk	SSS BGEM
Ind_0167	BGEM	(ONAVENO SON24/PHB47)/PHB47 #004-(2n)-004	Mexico	stiff stalk	NSS BGEM
Ind_0174	BGEM	(PATILLO GRANDE BOV649/PHB47)/PHB47 #003-(2n)-001	Bolivia	stiff stalk	SSS BGEM
Ind_0175	BGEM	(PATILLO GRANDE BOV649/PHB47)/PHB47 #006-(2n)-001	Bolivia	stiff stalk	SSS BGEM
Ind_0176	BGEM	(PHB47/PERLA ANC23)/PHB47 #002-(2n)-001	Peru	stiff stalk	SSS BGEM
Ind_0188	BGEM	(PHB47/PISAN BOV344)/PHB47 #003-(2n)-001	Bolivia	stiff stalk	SSS BGEM
Ind_0189	BGEM	(PHB47/PISAN BOV344)/PHB47 #005-(2n)-002	Bolivia	stiff stalk	SSS BGEM
Ind_0192	BGEM	(POJ CHICO BOV800/PHB47)/PHB47 #001-(2n)-001	Bolivia	stiff stalk	SSS BGEM
Ind_0193	BGEM	(POJ CHICO BOV800/PHB47)/PHB47 #002-(2n)-001	Bolivia	stiff stalk	SSS BGEM
Ind_0194	BGEM	(POJ CHICO BOV800/PHB47)/PHB47 #006-(2n)-003	Bolivia	stiff stalk	NSSS Ames
Ind_0195	BGEM	(POJ CHICO BOV800/PHB47)/PHB47 #006-(2n)-004	Bolivia	stiff stalk	NSS BGEM
Ind_0196	BGEM	(POJ CHICO BOV800/PHB47)/PHB47 #006-(2n)-005	Bolivia	stiff stalk	SSS BGEM
Ind_0197	BGEM	(POJ CHICO BOV800/PHB47)/PHB47 #006-(2n)-006	Bolivia	stiff stalk	SSS BGEM
Ind_0201	BGEM	((Semi dentado paulista - PAG I/PHB47 B)/PHB47)-(2n)-002	GEM	stiff stalk	SSS BGEM
Ind_0210	BGEM	((Vandeño - GRO 96/PHB47 B)/PHB47)-(2n)-001	GEM	stiff stalk	SSS BGEM
Ind_0217	BGEM	(YUCATAN TOL389 ICA/PHB47)/PHB47 #002-(2n)-001	GEM	stiff stalk	SSS BGEM
Ind_0218	BGEM	(YUCATAN TOL389 ICA/PHB47)/PHB47 #003-(2n)-001	GEM	stiff stalk	SSS BGEM
Ind_0219	BGEM	(YUCATAN TOL389 ICA/PHB47)/PHB47 #007-(2n)-001	GEM	stiff stalk	SSS BGEM
Ind_0224	BGEM	(YUNGUENO BOV362/PHB47)/PHB47 #001-(2n)-001	GEM	stiff stalk	SSS BGEM
Ind_0225	BGEM	(YUNQUILANO F AND ECU710/PHB47)/PHB47 #001-(2n)-001	GEM	stiff stalk	SSS BGEM
Ind_0226	BGEM	(YUNQUILANO F AND ECU710/PHB47)/PHB47 #007-(2n)-001	GEM	stiff stalk	SSS BGEM
Ind_0228	BGEM	((Chandelle - VEN 409/PHB47 B)/PHB47)-(2n)-001	GEM	stiff stalk	SSS BGEM
Ind_0229	BGEM	((Chirimito - VEN 703/PHB47 B)/PHB47)-(2n)-001	GEM	stiff stalk	SSS BGEM
Ind_0231	BGEM	((Culli - ARG 471/PHB47 B)/PHB47)-(2n)-001	GEM	stiff stalk	SSS BGEM
Ind_0232	BGEM	((Culli - ARG 471/PHB47 B)/PHB47)-(2n)-002	GEM	stiff stalk	SSS BGEM
Ind_0235	BGEM	((Tepecintle - GUA 65/PHB47 B)/PHB47)-(2n)-001	GEM	stiff stalk	SSS BGEM
Ind_0236	BGEM	((Capio rosado - ARG 460/PHB47 B)/PHB47)-(2n)-001	Argentina	stiff stalk	SSS BGEM
Ind_0237	BGEM	((Capio rosado - ARG 460/PHB47 B)/PHB47)-(2n)-002	Argentina	stiff stalk	SSS BGEM
Ind_0238	BGEM	((Cateto Nortista - GIN I/PHB47 B)/PHB47)-(2n)-002	Brazil (Guyana)	stiff stalk	SSS BGEM
Ind_0239	BGEM	((Chandelle - VEN 409/PHB47 B)/PHB47)-(2n)-002	GEM	stiff stalk	SSS BGEM
Ind_0240	BGEM	((PHB47/(Patillo - ECU 417/PHB47 B)-(2n)-001	GEM	stiff stalk	SSS BGEM
Ind_0241	BGEM	(ALTIPLANO BOV903/PHB47)/PHB47 #001-(2n)-001	GEM	stiff stalk	SSS BGEM
Ind_0243	BGEM	(ARAGUITO VEN678/PHB47)/PHB47 #003-(2n)-005	GEM	stiff stalk	SSS BGEM
Ind_0246	BGEM	(CAMELIA CHI411/PHB47)/PHB47 #005-(2n)-001	GEM	stiff stalk	SSS BGEM
Ind_0247	BGEM	((Canilla - VEN 693/PHB47 B)/PHB47)-(2n)-001	GEM	stiff stalk	SSS BGEM
Ind_0250	BGEM	((Comiteco - GUA 515/PHB47 B)/PHB47)-(2n)-001	GEM	stiff stalk	SSS BGEM
Ind_0251	BGEM	(CON NORT ZAC161/PHB47)/PHB47 #005-(2n)-007	GEM	stiff stalk	SSS BGEM
Ind_0253	BGEM	((Coroico blanco - BOV 406/PHB47 B)/PHB47)-(2n)-002	GEM	stiff stalk	SSS BGEM
Ind_0254	BGEM	((Cravo riograndense - RGS VII/PHB47 B)/PHB47)-(2n)-001	GEM	stiff stalk	SSS BGEM
Ind_0255	BGEM	(CRISTALINO AMAR AR21004/PHB47)/PHB47 #005-(2n)-001	GEM	stiff stalk	NSS BGEM
Ind_0259	BGEM	(DULCILLO DE NO SON57/PHB47)/PHB47 #005-(2n)-001	GEM	stiff stalk	SSS BGEM
Ind_0260	BGEM	(EARLY CARIBBEAN MAR9/PHB47)/PHB47 #002-(2n)-001	GEM	stiff stalk	SSS BGEM
Ind_0265	BGEM	((Huevito - VEN 396/PHB47 B)/PHB47)-(2n)-001	GEM	stiff stalk	SSS BGEM
Ind_0266	BGEM	((Huevito - VEN 396/PHB47 B)/PHB47)-(2n)-002	GEM	stiff stalk	SSS BGEM
Ind_0267	BGEM	(KARAPAMPA BOV978/PHB47)/PHB47 #005-(2n)-001	GEM	stiff stalk	SSS BGEM
Ind_0271	BGEM	(MONTANA NAR625/PHB47)/PHB47 #006-(2n)-002	GEM	stiff stalk	SSS BGEM
Ind_0272	BGEM	(MORADO BOV567/PHB47)/PHB47 #005-(2n)-001	GEM	stiff stalk	SSS BGEM
Ind_0273	BGEM	(MORADO BOV567/PHB47)/PHB47 #006-(2n)-001	GEM	stiff stalk	SSS BGEM
Ind_0274	BGEM	(MORADO CANTENO LIM55/PHB47)/PHB47 #002-(2n)-003	GEM	stiff stalk	SSS BGEM

Table S1. The 679 maize lines comprising the Combined panel, pedigree, breeding program, heterotic group and subpopulation by fasStructure.

Accession	Panel	pedigree	Breeding program	Heterotic Group	fastStructure
Ind_0276	BGEM	((Nineulo - BOV 1088/PHB47 B)/PHB47)-(2n)-001	GEM	stiff stalk	SSS BGEM
Ind_0277	BGEM	((Oke - ARG 539/PHB47 B)/PHB47)-(2n)-001	GEM	stiff stalk	SSS BGEM
Ind_0281	BGEM	(POJ CHICO BOV800/PHB47)/PHB47 #001-(2n)-002	GEM	stiff stalk	NSS BGEM
Ind_0283	BGEM	((Rabo De Zorro - ANC 325/PHB47 B)/PHB47)-(2n)-001	GEM	stiff stalk	SSS BGEM
Ind_0284	BGEM	((Rabo De Zorro - ANC 325/PHB47 B)/PHB47)-(2n)-003	GEM	stiff stalk	SSS BGEM
Ind_0285	BGEM	(RATON CHH191/PHB47)/PHB47 #003-(2n)-001	GEM	stiff stalk	SSS BGEM
Ind_0286	BGEM	((Reventador - NAY 39/PHB47 B)/PHB47)-(2n)-001	GEM	stiff stalk	NSS BGEM
Ind_0287	BGEM	((Reventador - NAY 39/PHB47 B)/PHB47)-(2n)-002	GEM	stiff stalk	NSSS Ames
Ind_0288	BGEM	((Reventador - NAY 39/PHB47 B)/PHB47)-(2n)-003	GEM	stiff stalk	SSS BGEM
Ind_0290	BGEM	((Semi dentado paulista - PAG I/PHB47 B)/PHB47)-(2n)-003	GEM	stiff stalk	SSS BGEM
Ind_0291	BGEM	((Semi dentado paulista - PAG I/PHB47 B)/PHB47)-(2n)-004	GEM	stiff stalk	SSS BGEM
Ind_0294	BGEM	((Tuxpeño - GUA 456/PHB47 B)/PHB47)-(2n)-002	GEM	stiff stalk	SSS BGEM
Ind_0295	BGEM	((Tuxpeño - GUA 456/PHB47 B)/PHB47)-(2n)-003	GEM	stiff stalk	SSS BGEM
Ind_0301	BGEM	PHB47	GEM	stiff stalk	SSS BGEM
Ind_0634	Ames	LH39	ExPVP	non-stiff stalk	NSSS Ames
Ind_0640	Ames	LH123HT	ExPVP	non-stiff stalk	NSSS Ames
Ind_0643	Ames	PHG72	ExPVP	non-stiff stalk	NSSS Ames
Ind_0644	Ames	PHG84	ExPVP	non-stiff stalk	NSSS Ames
Ind_0645	Ames	PHZ51	ExPVP	non-stiff stalk	NSS BGEM
Ind_0646	Ames	LH156	ExPVP	non-stiff stalk	NSSS Ames
Ind_0647	Ames	78371A	ExPVP	non-stiff stalk	NSSS Ames
Ind_0649	Ames	LH59	ExPVP	non-stiff stalk	NSSS Ames
Ind_0653	Ames	LH65	ExPVP	non-stiff stalk	NSSS Ames
Ind_0654	Ames	PHT77	ExPVP	non-stiff stalk	NSSS Ames
Ind_0655	Ames	PHV63	ExPVP	non-stiff stalk	NSSS Ames
Ind_0656	Ames	PHW65	ExPVP	non-stiff stalk	NSSS Ames
Ind_0657	Ames	6M502	ExPVP	non-stiff stalk	NSSS Ames
Ind_0658	Ames	PHT60	ExPVP	non-stiff stalk	NSSS Ames
Ind_0661	Ames	WIL901	ExPVP	non-stiff stalk	NSSS Ames
Ind_0662	Ames	WIL903	ExPVP	non-stiff stalk	NSSS Ames
Ind_0664	Ames	L127	ExPVP	non-stiff stalk	NSSS Ames
Ind_0665	Ames	L139	ExPVP	non-stiff stalk	NSSS Ames
Ind_0669	Ames	PHN73	ExPVP	non-stiff stalk	NSSS Ames
Ind_0672	Ames	PHT22	ExPVP	non-stiff stalk	NSSS Ames
Ind_0318	Ames	H107	Indiana	non-stiff stalk	NSSS Ames
Ind_0319	Ames	H95	Indiana	non-stiff stalk	NSSS Ames
Ind_0323	Ames	H25W	Indiana	non-stiff stalk	NSSS Ames
Ind_0359	Ames	H22w	Indiana	non-stiff stalk	NSSS Ames
Ind_0415	Ames	H116	Indiana	non-stiff stalk	NSSS Ames
Ind_0588	Ames	H126W	Indiana	non-stiff stalk	NSSS Ames
Ind_0338	Ames	MS68	Michigan	non-stiff stalk	NSSS Ames
Ind_0348	Ames	MS222	Michigan	non-stiff stalk	SSS Ames
Ind_0349	Ames	MS223	Michigan	non-stiff stalk	NSSS Ames
Ind_0350	Ames	MS224	Michigan	non-stiff stalk	NSSS Ames
Ind_0412	Ames	Mo307ae	Missouri	non-stiff stalk	NSSS Ames
Ind_0567	Ames	Mo20W	Missouri	non-stiff stalk	NSSS Ames
Ind_0578	Ames	Mo17	Missouri	non-stiff stalk	NSSS Ames
Ind_0587	Ames	Mo47	Missouri	non-stiff stalk	NSSS Ames
Ind_0380	Ames	NC260	North Carolina	non-stiff stalk	NSSS Ames
Ind_0306	Ames	Va35C	Other	non-stiff stalk	NSSS Ames
Ind_0307	Ames	Va36A	Other	non-stiff stalk	NSSS Ames
Ind_0313	Ames	Va59	Other	non-stiff stalk	NSSS Ames
Ind_0317	Ames	C123	Other	non-stiff stalk	NSSS Ames
Ind_0322	Ames	Va22	Other	non-stiff stalk	NSSS Ames
Ind_0352	Ames	Va99	Other	non-stiff stalk	NSSS Ames
Ind_0396	Ames	Oh40B	Other	non-stiff stalk	NSSS Ames
Ind_0551	Ames	Pa356	Other	non-stiff stalk	NSSS Ames
Ind_0552	Ames	Pa376	Other	non-stiff stalk	NSSS Ames
Ind_0595	Ames	Va35	Other	non-stiff stalk	NSSS Ames
Ind_0637	Ames	PHG35	Other	non-stiff stalk	NSSS Ames
Ind_0399	Ames	W22	Wisconsin	non-stiff stalk	NSSS Ames
Ind_0592	Ames	HP301	Indiana	popcorn	NSSS Ames
Ind_0383	Ames	SA24	Other	popcorn	NSSS Ames
Ind_0413	Ames	IADS61	Other	popcorn	NSSS Ames
Ind_0530	Ames	IADS43-W	Other	popcorn	NSSS Ames
Ind_0554	Ames	LH195	ExPVP	stiff stalk	SSS Ames
Ind_0555	Ames	LH205	ExPVP	stiff stalk	SSS Ames
Ind_0557	Ames	LH220Ht	ExPVP	stiff stalk	SSS Ames
Ind_0561	Ames	LH202	ExPVP	stiff stalk	SSS Ames
Ind_0566	Ames	LH208	ExPVP	stiff stalk	SSS Ames
Ind_0580	Ames	ICI441	ExPVP	stiff stalk	SSS Ames
Ind_0632	Ames	LP1CMSHT	ExPVP	stiff stalk	SSS Ames

Table S1. The 679 maize lines comprising the Combined panel, pedigree, breeding program, heterotic group and subpopulation by fasStructure.

Accession	Panel	pedigree	Breeding program	Heterotic Group	fastStructure
Ind_0633	Ames	FR19	ExPVP	stiff stalk	NSSS Ames
Ind_0635	Ames	LH74	ExPVP	stiff stalk	SSS Ames
Ind_0636	Ames	FAPW	ExPVP	stiff stalk	SSS Ames
Ind_0638	Ames	B47	ExPVP	stiff stalk	SSS BGEM
Ind_0639	Ames	G80	ExPVP	stiff stalk	NSSS Ames
Ind_0641	Ames	78004	ExPVP	stiff stalk	SSS Ames
Ind_0642	Ames	78002A	ExPVP	stiff stalk	SSS Ames
Ind_0648	Ames	PB80	ExPVP	stiff stalk	SSS Ames
Ind_0650	Ames	PHK29	ExPVP	stiff stalk	SSS Ames
Ind_0652	Ames	LH149	ExPVP	stiff stalk	SSS Ames
Ind_0659	Ames	H8431	ExPVP	stiff stalk	SSS Ames
Ind_0668	Ames	PHM10	ExPVP	stiff stalk	NSSS Ames
Ind_0670	Ames	PHP55	ExPVP	stiff stalk	NSSS Ames
Ind_0673	Ames	PHV37	ExPVP	stiff stalk	SSS Ames
Ind_0572	Ames	H111	Indiana	stiff stalk	SSS Ames
Ind_0590	Ames	H105W	Indiana	stiff stalk	SSS Ames
Ind_0591	Ames	H84	Indiana	stiff stalk	SSS BGEM
Ind_0569	Ames	B73	Iowa	stiff stalk	SSS Ames
Ind_0345	Ames	MS132	Michigan	stiff stalk	NSSS Ames
Ind_0316	Ames	A634	Minnesota	stiff stalk	SSS Ames
Ind_0331	Ames	A633	Minnesota	stiff stalk	SSS Ames
Ind_0414	Ames	A657	Minnesota	stiff stalk	NSSS Ames
Ind_0594	Ames	A632	Minnesota	stiff stalk	SSS Ames
Ind_0388	Ames	Mo401	Missouri	stiff stalk	SSS BGEM
Ind_0390	Ames	N7AGoodman-Buckler	Nebraska	stiff stalk	NSSS Ames
Ind_0678	Ames	N7A	Nebraska	stiff stalk	NSS BGEM
Ind_0545	Ames	NC252	North Carolina	stiff stalk	SSS Ames
Ind_0546	Ames	NC254	North Carolina	stiff stalk	SSS Ames
Ind_0547	Ames	NC256	North Carolina	stiff stalk	SSS Ames
Ind_0573	Ames	NC250	North Carolina	stiff stalk	SSS BGEM
Ind_0305	Ames	38-11RPARENTHB19INB	Other	stiff stalk	NSSS Ames
Ind_0315	Ames	Wf9	Other	stiff stalk	NSSS Ames
Ind_0353	Ames	Pa91HT1	Other	stiff stalk	NSSS Ames
Ind_0356	Ames	WX38-11	Other	stiff stalk	NSSS Ames
Ind_0548	Ames	Pa879	Other	stiff stalk	NSSS Ames
Ind_0539	Ames	A641N	South Africa	stiff stalk	SSS Ames
Ind_0400	Ames	W23	Wisconsin	stiff stalk	NSSS Ames
Ind_0324	Ames	C15	Other	sweet corn	NSSS Ames
Ind_0325	Ames	C18	Other	sweet corn	NSSS Ames
Ind_0385	Ames	Il731a	Other	sweet corn	NSSS Ames
Ind_0386	Ames	Il767b	Other	sweet corn	NSSS Ames
Ind_0387	Ames	P39Goodman-Buckler	Other	sweet corn	NSSS Ames
Ind_0589	Ames	C13	Other	sweet corn	NSSS Ames
Ind_0628	Ames	CML247	Mexico	tropical	NSSS Ames
Ind_0575	Ames	89199	Other	tropical	NSSS Ames
Ind_0377	Ames	Ki11	Thailand	tropical	NSSS Ames
Ind_0378	Ames	Ki21	Thailand	tropical	NSSS Ames
Ind_0473	Ames	INBRED2-687	Argentina	unclassified	NSSS Ames
Ind_0474	Ames	INBRED1-1265	Argentina	unclassified	NSSS Ames
Ind_0475	Ames	INBRED19-86	Argentina	unclassified	NSSS Ames
Ind_0476	Ames	INBRED34-1141	Argentina	unclassified	NSSS Ames
Ind_0477	Ames	INBRED34-1196	Argentina	unclassified	NSSS Ames
Ind_0482	Ames	4F-35BK	Argentina	unclassified	NSSS Ames
Ind_0483	Ames	4F-203AM6	Argentina	unclassified	NSSS Ames
Ind_0484	Ames	4F-234BX4	Argentina	unclassified	NSSS Ames
Ind_0485	Ames	4F-285TX15	Argentina	unclassified	NSSS Ames
Ind_0486	Ames	4F-306108	Argentina	unclassified	NSSS Ames
Ind_0487	Ames	4F-345CN12	Argentina	unclassified	NSSS Ames
Ind_0416	Ames	AusTRCF305819	Australia	unclassified	SSS Ames
Ind_0417	Ames	AusTRCF305822	Australia	unclassified	NSSS Ames
Ind_0418	Ames	AusTRCF305833	Australia	unclassified	SSS Ames
Ind_0419	Ames	AusTRCF305835	Australia	unclassified	SSS Ames
Ind_0420	Ames	AusTRCF305836	Australia	unclassified	SSS Ames
Ind_0421	Ames	AusTRCF305839	Australia	unclassified	SSS Ames
Ind_0422	Ames	AusTRCF305849	Australia	unclassified	NSSS Ames
Ind_0423	Ames	AusTRCF306065	Australia	unclassified	NSSS Ames
Ind_0424	Ames	AusTRCF306235	Australia	unclassified	SSS Ames
Ind_0425	Ames	AusTRCF306236	Australia	unclassified	NSSS Ames
Ind_0426	Ames	AusTRCF306237	Australia	unclassified	NSSS Ames
Ind_0427	Ames	AusTRCF306239	Australia	unclassified	NSSS Ames
Ind_0428	Ames	AusTRCF306240	Australia	unclassified	NSSS Ames
Ind_0429	Ames	AusTRCF306241	Australia	unclassified	NSSS Ames

Table S1. The 679 maize lines comprising the Combined panel, pedigree, breeding program, heterotic group and subpopulation by fasStructure.

Accession	Panel	pedigree	Breeding program	Heterotic Group	fastStructure
Ind_0430	Ames	AusTRCF306244	Australia	unclassified	NSSS Ames
Ind_0431	Ames	AusTRCF306254	Australia	unclassified	NSSS Ames
Ind_0432	Ames	AusTRCF306257	Australia	unclassified	NSSS Ames
Ind_0433	Ames	AusTRCF306261	Australia	unclassified	NSSS Ames
Ind_0434	Ames	AusTRCF306264	Australia	unclassified	NSSS Ames
Ind_0435	Ames	AusTRCF306273	Australia	unclassified	NSSS Ames
Ind_0436	Ames	AusTRCF306274	Australia	unclassified	NSSS Ames
Ind_0437	Ames	AusTRCF306276	Australia	unclassified	NSSS Ames
Ind_0438	Ames	AusTRCF306278	Australia	unclassified	NSSS Ames
Ind_0439	Ames	AusTRCF306280	Australia	unclassified	NSSS Ames
Ind_0440	Ames	AusTRCF306281	Australia	unclassified	NSSS Ames
Ind_0441	Ames	AusTRCF306282	Australia	unclassified	NSSS Ames
Ind_0442	Ames	AusTRCF306285	Australia	unclassified	NSSS Ames
Ind_0443	Ames	AusTRCF306287	Australia	unclassified	NSSS Ames
Ind_0444	Ames	AusTRCF306290	Australia	unclassified	NSSS Ames
Ind_0445	Ames	AusTRCF306293	Australia	unclassified	NSSS Ames
Ind_0446	Ames	AusTRCF306296	Australia	unclassified	NSSS Ames
Ind_0447	Ames	AusTRCF306303	Australia	unclassified	SSS Ames
Ind_0448	Ames	AusTRCF306304	Australia	unclassified	SSS Ames
Ind_0449	Ames	AusTRCF306306	Australia	unclassified	SSS Ames
Ind_0450	Ames	AusTRCF306307	Australia	unclassified	SSS Ames
Ind_0451	Ames	AusTRCF306308	Australia	unclassified	SSS Ames
Ind_0452	Ames	AusTRCF306309	Australia	unclassified	SSS Ames
Ind_0453	Ames	AusTRCF306310	Australia	unclassified	SSS Ames
Ind_0454	Ames	AusTRCF306321	Australia	unclassified	NSSS Ames
Ind_0455	Ames	AusTRCF306323	Australia	unclassified	NSSS Ames
Ind_0456	Ames	AusTRCF306324	Australia	unclassified	NSSS Ames
Ind_0457	Ames	AusTRCF306333	Australia	unclassified	SSS Ames
Ind_0458	Ames	AusTRCF306335	Australia	unclassified	NSSS Ames
Ind_0459	Ames	AusTRCF306336	Australia	unclassified	NSSS Ames
Ind_0460	Ames	AusTRCF306337	Australia	unclassified	NSSS Ames
Ind_0461	Ames	AusTRCF306343	Australia	unclassified	NSSS Ames
Ind_0462	Ames	AusTRCF306344	Australia	unclassified	SSS Ames
Ind_0463	Ames	AusTRCF306345	Australia	unclassified	NSSS Ames
Ind_0464	Ames	AusTRCF306347	Australia	unclassified	SSS Ames
Ind_0465	Ames	AusTRCF306348	Australia	unclassified	SSS Ames
Ind_0466	Ames	AusTRCF306350	Australia	unclassified	NSSS Ames
Ind_0467	Ames	AusTRCF306352	Australia	unclassified	NSSS Ames
Ind_0303	Ames	Bei10=North10	China	unclassified	NSSS Ames
Ind_0531	Ames	CHI-41	China	unclassified	NSSS Ames
Ind_0532	Ames	CHAN11INBRED	China	unclassified	NSSS Ames
Ind_0533	Ames	BAITOUSHUANGIN.JI095	China	unclassified	NSSS Ames
Ind_0556	Ames	LH206	ExPVP	unclassified	SSS Ames
Ind_0579	Ames	ICI193	ExPVP	unclassified	NSSS Ames
Ind_0581	Ames	ICI740	ExPVP	unclassified	SSS Ames
Ind_0582	Ames	ICI893	ExPVP	unclassified	SSS Ames
Ind_0651	Ames	740	ExPVP	unclassified	NSSS Ames
Ind_0660	Ames	WIL900	ExPVP	unclassified	NSSS Ames
Ind_0663	Ames	J8606	ExPVP	unclassified	NSSS Ames
Ind_0666	Ames	W8555	ExPVP	unclassified	SSS Ames
Ind_0667	Ames	PHK35	ExPVP	unclassified	SSS Ames
Ind_0510	Ames	FV181	France	unclassified	NSSS Ames
Ind_0511	Ames	FC46	France	unclassified	NSSS Ames
Ind_0320	Ames	R168	Illinois	unclassified	NSSS Ames
Ind_0398	Ames	R221	Illinois	unclassified	NSSS Ames
Ind_0405	Ames	R30	Illinois	unclassified	NSSS Ames
Ind_0406	Ames	R105	Illinois	unclassified	NSSS Ames
Ind_0596	Ames	R230	Illinois	unclassified	SSS Ames
Ind_0357	Ames	H14	Indiana	unclassified	NSSS Ames
Ind_0358	Ames	H19	Indiana	unclassified	NSSS Ames
Ind_0360	Ames	H23w	Indiana	unclassified	NSSS Ames
Ind_0361	Ames	H26w	Indiana	unclassified	NSSS Ames
Ind_0362	Ames	H27w	Indiana	unclassified	NSSS Ames
Ind_0363	Ames	H29w	Indiana	unclassified	NSSS Ames
Ind_0364	Ames	H41	Indiana	unclassified	NSSS Ames
Ind_0365	Ames	H50	Indiana	unclassified	NSSS Ames
Ind_0366	Ames	H52	Indiana	unclassified	NSSS Ames
Ind_0367	Ames	H55	Indiana	unclassified	NSSS Ames
Ind_0368	Ames	H59	Indiana	unclassified	NSSS Ames
Ind_0369	Ames	H88	Indiana	unclassified	NSSS Ames
Ind_0563	Ames	HP72-11	Indiana	unclassified	NSSS Ames
Ind_0570	Ames	H102	Indiana	unclassified	NSSS Ames

Table S1. The 679 maize lines comprising the Combined panel, pedigree, breeding program, heterotic group and subpopulation by fasStructure.

Accession	Panel	pedigree	Breeding program	Heterotic Group	fastStructure
Ind_0571	Ames	H103	Indiana	unclassified	NSSS Ames
Ind_0411	Ames	B10	Iowa	unclassified	NSSS Ames
Ind_0568	Ames	B46	Iowa	unclassified	NSSS Ames
Ind_0337	Ames	MS4	Michigan	unclassified	NSSS Ames
Ind_0339	Ames	MS72	Michigan	unclassified	NSSS Ames
Ind_0340	Ames	MS76	Michigan	unclassified	SSS Ames
Ind_0341	Ames	MS78	Michigan	unclassified	NSSS Ames
Ind_0342	Ames	MS80	Michigan	unclassified	NSSS Ames
Ind_0343	Ames	MS91	Michigan	unclassified	NSSS Ames
Ind_0344	Ames	MS106	Michigan	unclassified	NSSS Ames
Ind_0346	Ames	MS141	Michigan	unclassified	NSSS Ames
Ind_0347	Ames	MS198	Michigan	unclassified	NSSS Ames
Ind_0351	Ames	MS226	Michigan	unclassified	NSSS Ames
Ind_0326	Ames	A265	Minnesota	unclassified	NSSS Ames
Ind_0327	Ames	A286	Minnesota	unclassified	NSSS Ames
Ind_0328	Ames	A427	Minnesota	unclassified	NSSS Ames
Ind_0329	Ames	A617	Minnesota	unclassified	NSSS Ames
Ind_0330	Ames	A630	Minnesota	unclassified	NSSS Ames
Ind_0332	Ames	A636	Minnesota	unclassified	SSS Ames
Ind_0333	Ames	A637	Minnesota	unclassified	NSSS Ames
Ind_0334	Ames	A643	Minnesota	unclassified	NSSS Ames
Ind_0335	Ames	A644	Minnesota	unclassified	NSSS Ames
Ind_0336	Ames	A645	Minnesota	unclassified	NSSS Ames
Ind_0593	Ames	A554	Minnesota	unclassified	NSSS Ames
Ind_0370	Ames	Mo41	Missouri	unclassified	NSSS Ames
Ind_0379	Ames	MoG	Missouri	unclassified	NSSS Ames
Ind_0389	Ames	Mo402	Missouri	unclassified	NSSS Ames
Ind_0391	Ames	Mo30W	Missouri	unclassified	NSSS Ames
Ind_0392	Ames	Mo37	Missouri	unclassified	NSSS Ames
Ind_0393	Ames	Mo39	Missouri	unclassified	NSSS Ames
Ind_0576	Ames	MO1W	Missouri	unclassified	NSSS Ames
Ind_0577	Ames	Mo2RF	Missouri	unclassified	NSSS Ames
Ind_0562	Ames	NP87	Nebraska	unclassified	NSSS Ames
Ind_0585	Ames	N199	Nebraska	unclassified	NSSS Ames
Ind_0599	Ames	N501	Nebraska	unclassified	NSSS Ames
Ind_0600	Ames	N502	Nebraska	unclassified	SSS Ames
Ind_0601	Ames	N509	Nebraska	unclassified	NSSS Ames
Ind_0602	Ames	N510	Nebraska	unclassified	SSS Ames
Ind_0603	Ames	N511	Nebraska	unclassified	NSSS Ames
Ind_0604	Ames	N512	Nebraska	unclassified	NSSS Ames
Ind_0605	Ames	N514	Nebraska	unclassified	NSSS Ames
Ind_0606	Ames	N515	Nebraska	unclassified	NSSS Ames
Ind_0607	Ames	N516	Nebraska	unclassified	NSSS Ames
Ind_0608	Ames	N517	Nebraska	unclassified	NSSS Ames
Ind_0609	Ames	N518	Nebraska	unclassified	NSSS Ames
Ind_0610	Ames	N521	Nebraska	unclassified	NSSS Ames
Ind_0611	Ames	N523	Nebraska	unclassified	NSSS Ames
Ind_0612	Ames	N524	Nebraska	unclassified	NSSS Ames
Ind_0613	Ames	N525	Nebraska	unclassified	SSS Ames
Ind_0614	Ames	N526	Nebraska	unclassified	NSSS Ames
Ind_0615	Ames	N528	Nebraska	unclassified	NSSS Ames
Ind_0616	Ames	N529	Nebraska	unclassified	NSSS Ames
Ind_0617	Ames	N530	Nebraska	unclassified	NSSS Ames
Ind_0618	Ames	N532	Nebraska	unclassified	NSSS Ames
Ind_0619	Ames	N533	Nebraska	unclassified	NSSS Ames
Ind_0620	Ames	N534	Nebraska	unclassified	NSSS Ames
Ind_0621	Ames	N535	Nebraska	unclassified	NSSS Ames
Ind_0622	Ames	N538	Nebraska	unclassified	NSSS Ames
Ind_0623	Ames	N541	Nebraska	unclassified	NSSS Ames
Ind_0624	Ames	N542	Nebraska	unclassified	NSSS Ames
Ind_0625	Ames	N543	Nebraska	unclassified	NSSS Ames
Ind_0626	Ames	N544	Nebraska	unclassified	NSSS Ames
Ind_0627	Ames	N209	Nebraska	unclassified	NSSS Ames
Ind_0629	Ames	N216	Nebraska	unclassified	NSSS Ames
Ind_0630	Ames	N218	Nebraska	unclassified	SSS Ames
Ind_0631	Ames	N546	Nebraska	unclassified	NSSS Ames
Ind_0671	Ames	N552	Nebraska	unclassified	NSSS Ames
Ind_0674	Ames	N527	Nebraska	unclassified	NSSS Ames
Ind_0381	Ames	NC306	North Carolina	unclassified	SSS Ames
Ind_0382	Ames	NC308	North Carolina	unclassified	SSS Ames
Ind_0553	Ames	NC262	North Carolina	unclassified	NSSS Ames
Ind_0409	Ames	ND408	North Dakota	unclassified	NSSS Ames

Table S1. The 679 maize lines comprising the Combined panel, pedigree, breeding program, heterotic group and subpopulation by fasStructure.

Accession	Panel	pedigree	Breeding program	Heterotic Group	fastStructure
Ind_0410	Ames	ND480	North Dakota	unclassified	NSSS Ames
Ind_0371	Ames	CH705-8	Ontario	unclassified	NSSS Ames
Ind_0372	Ames	CH711-10	Ontario	unclassified	NSSS Ames
Ind_0373	Ames	CH732-12	Ontario	unclassified	SSS Ames
Ind_0374	Ames	CH741-6	Ontario	unclassified	SSS Ames
Ind_0375	Ames	CH701-30	Ontario	unclassified	NSSS Ames
Ind_0304	Ames	52220	Other	unclassified	NSSS Ames
Ind_0308	Ames	VaW6	Other	unclassified	NSSS Ames
Ind_0309	Ames	Va24	Other	unclassified	NSSS Ames
Ind_0310	Ames	Va38	Other	unclassified	NSSS Ames
Ind_0311	Ames	Va39	Other	unclassified	NSSS Ames
Ind_0312	Ames	Va46	Other	unclassified	NSSS Ames
Ind_0314	Ames	Va91	Other	unclassified	NSSS Ames
Ind_0321	Ames	Tx303	Other	unclassified	NSSS Ames
Ind_0354	Ames	P8	Other	unclassified	NSSS Ames
Ind_0355	Ames	CI20	Other	unclassified	NSSS Ames
Ind_0376	Ames	K148	Other	unclassified	NSSS Ames
Ind_0384	Ames	Va85	Other	unclassified	NSSS Ames
Ind_0394	Ames	K150	Other	unclassified	NSSS Ames
Ind_0395	Ames	K41	Other	unclassified	NSSS Ames
Ind_0397	Ames	OH84	Other	unclassified	NSSS Ames
Ind_0403	Ames	SD10	Other	unclassified	NSSS Ames
Ind_0404	Ames	L	Other	unclassified	NSSS Ames
Ind_0407	Ames	90	Other	unclassified	NSSS Ames
Ind_0408	Ames	5120B	Other	unclassified	NSSS Ames
Ind_0468	Ames	INBRED378	Other	unclassified	NSSS Ames
Ind_0469	Ames	INBRED624	Other	unclassified	NSSS Ames
Ind_0470	Ames	INBRED45	Other	unclassified	NSSS Ames
Ind_0472	Ames	INBRED141	Other	unclassified	NSSS Ames
Ind_0478	Ames	INBRED305	Other	unclassified	NSSS Ames
Ind_0479	Ames	INBRED309	Other	unclassified	NSSS Ames
Ind_0480	Ames	INBRED321	Other	unclassified	NSSS Ames
Ind_0481	Ames	INBRED334	Other	unclassified	NSSS Ames
Ind_0488	Ames	NY3NevehYaar	Other	unclassified	NSSS Ames
Ind_0489	Ames	NY159NevehYaar	Other	unclassified	NSSS Ames
Ind_0490	Ames	NY166NevehYaar	Other	unclassified	NSSS Ames
Ind_0491	Ames	NY188NevehYaar	Other	unclassified	NSSS Ames
Ind_0492	Ames	NY318NeveyYaar	Other	unclassified	NSSS Ames
Ind_0493	Ames	NY364NevehYaar	Other	unclassified	NSSS Ames
Ind_0494	Ames	NY643NevehYaar	Other	unclassified	NSSS Ames
Ind_0495	Ames	NY971NevehYaar	Other	unclassified	NSSS Ames
Ind_0496	Ames	NY1000NevehYaar	Other	unclassified	NSSS Ames
Ind_0512	Ames	T8445INBRED	Other	unclassified	NSSS Ames
Ind_0522	Ames	TN53-1-2	Other	unclassified	NSSS Ames
Ind_0523	Ames	NY121NevehYaar	Other	unclassified	NSSS Ames
Ind_0524	Ames	NY123NevehYaar	Other	unclassified	NSSS Ames
Ind_0525	Ames	G3T5	Other	unclassified	NSSS Ames
Ind_0526	Ames	G22T122	Other	unclassified	NSSS Ames
Ind_0527	Ames	G14T133	Other	unclassified	NSSS Ames
Ind_0528	Ames	G15T134	Other	unclassified	NSSS Ames
Ind_0529	Ames	T141	Other	unclassified	NSSS Ames
Ind_0540	Ames	4581INBRED	Other	unclassified	NSSS Ames
Ind_0541	Ames	M6411	Other	unclassified	NSSS Ames
Ind_0542	Ames	M6415	Other	unclassified	NSSS Ames
Ind_0543	Ames	M6421	Other	unclassified	NSSS Ames
Ind_0544	Ames	SD42	Other	unclassified	NSSS Ames
Ind_0549	Ames	Pa880	Other	unclassified	NSSS Ames
Ind_0550	Ames	SD46	Other	unclassified	NSSS Ames
Ind_0558	Ames	SD53	Other	unclassified	SSS Ames
Ind_0559	Ames	SD106	Other	unclassified	NSSS Ames
Ind_0560	Ames	SD108	Other	unclassified	SSS Ames
Ind_0564	Ames	Va4	Other	unclassified	NSSS Ames
Ind_0565	Ames	Va5	Other	unclassified	NSSS Ames
Ind_0574	Ames	DE811	Other	unclassified	NSSS Ames
Ind_0583	Ames	NQ508	Other	unclassified	NSSS Ames
Ind_0584	Ames	NYRD4058	Other	unclassified	NSSS Ames
Ind_0586	Ames	Oh599	Other	unclassified	NSSS Ames
Ind_0597	Ames	Hi27	Other	unclassified	NSSS Ames
Ind_0598	Ames	Hi34	Other	unclassified	NSSS Ames
Ind_0675	Ames	DE1	Other	unclassified	NSSS Ames
Ind_0676	Ames	SD40	Other	unclassified	NSSS Ames
Ind_0677	Ames	SD41	Other	unclassified	NSSS Ames

Table S1. The 679 maize lines comprising the Combined panel, pedigree, breeding program, heterotic group and subpopulation by fasStructure.

Accession	Panel	pedigree	Breeding program	Heterotic Group	fastStructure
Ind_0679	Ames	Tx714	Other	unclassified	SSS Ames
Ind_0471	Ames	INBREDA-243-1	South Africa	unclassified	NSSS Ames
Ind_0497	Ames	A14INBREDPOTCHEFSTROOMPEARL	South Africa	unclassified	NSSS Ames
Ind_0498	Ames	A15-1INBREDPOTCHEFSTROOMPEARL	South Africa	unclassified	NSSS Ames
Ind_0499	Ames	A16-3-2INBREDPOTCHEFSTROOMPEARL	South Africa	unclassified	NSSS Ames
Ind_0500	Ames	E205-1-1-1INBREDS5SYN.ANVELD	South Africa	unclassified	NSSS Ames
Ind_0501	Ames	A415-1-3INBRED	South Africa	unclassified	NSSS Ames
Ind_0502	Ames	A436-1INBRED	South Africa	unclassified	NSSS Ames
Ind_0503	Ames	E683-1-2-1S5INBRED	South Africa	unclassified	NSSS Ames
Ind_0504	Ames	E684-1-1-1S5INBRED	South Africa	unclassified	NSSS Ames
Ind_0505	Ames	A242-2S10INBREDPERUVIAN	South Africa	unclassified	NSSS Ames
Ind_0506	Ames	A243-1-2S10INBREDPERUVIAN	South Africa	unclassified	NSSS Ames
Ind_0507	Ames	A256-1S10INBREDPERUVIAN	South Africa	unclassified	NSSS Ames
Ind_0508	Ames	A302-1-2S10INBREDSERVENTINA	South Africa	unclassified	NSSS Ames
Ind_0509	Ames	C410-1F11INBREDHOTNOTCROSSES	South Africa	unclassified	NSSS Ames
Ind_0534	Ames	A14NW	South Africa	unclassified	NSSS Ames
Ind_0535	Ames	A57N	South Africa	unclassified	NSSS Ames
Ind_0536	Ames	A178N	South Africa	unclassified	NSSS Ames
Ind_0537	Ames	A579N	South Africa	unclassified	NSSS Ames
Ind_0538	Ames	A622N	South Africa	unclassified	NSSS Ames
Ind_0513	Ames	NO.1004INBRED	Spain	unclassified	NSSS Ames
Ind_0514	Ames	NO.1019INBRED	Spain	unclassified	NSSS Ames
Ind_0515	Ames	NO.1032INBRED	Spain	unclassified	NSSS Ames
Ind_0516	Ames	NO.1037INBRED	Spain	unclassified	NSSS Ames
Ind_0517	Ames	NO.1049INBRED	Spain	unclassified	NSSS Ames
Ind_0518	Ames	NO.1068INBRED	Spain	unclassified	NSSS Ames
Ind_0519	Ames	NO.1070INBRED	Spain	unclassified	NSSS Ames
Ind_0520	Ames	NO.1174INBRED	Spain	unclassified	NSSS Ames
Ind_0521	Ames	NO.1201INBRED	Spain	unclassified	NSSS Ames
Ind_0401	Ames	W24	Wisconsin	unclassified	NSSS Ames
Ind_0402	Ames	W32	Wisconsin	unclassified	NSSS Ames
